# Evolution of the Polyphenol and Terpene Content, Antioxidant Activity and Plant Morphology of Eight Different Fiber-Type Cultivars of *Cannabis sativa* L. Cultivated at Three Sowing Densities

**DOI:** 10.3390/plants9121740

**Published:** 2020-12-09

**Authors:** Amandine André, Marianne Leupin, Markus Kneubühl, Vasilisa Pedan, Irene Chetschik

**Affiliations:** Department of Life Sciences and Facility Management, Zurich University of Applied Sciences, 8820 Wädenswil, Switzerland; leup@zhaw.ch (M.L.); kneu@zhaw.ch (M.K.); pedn@zhaw.ch (V.P.); chet@zhaw.ch (I.C.)

**Keywords:** fibre-type hemp, polyphenols, flavonoids, terpenes, plant morphology

## Abstract

The chemical composition of the inflorescences of eight different fibre-type *Cannabis sativa* L. cultivars grown in Switzerland was monitored for different sowing densities over the season 2019. HPLC-MS, GC-MS and GC-FID, as well as spectrophotometric techniques were used to measure the total phenolic content (TPC) and the antioxidative activity of the inflorescence extracts, and to characterise and quantify the flavonoids and terpenes produced by the different cultivars over different sowing densities from July to September 2019. The main finding of the present study is that the TPC, as well as the individual flavonoids and terpenes, were mainly influenced by the harvest period and the phenological stage of the plant. The content of polyphenols and flavonoids decrease during the flower development for all cultivars studied. The terpene content increased with maturation. The monoterpenes/sesquiterpenes ratio also changed between the early flowering (majority of sesquiterpenes) and the end of flowering (majority of monoterpenes). The sowing density showed an impact on plant morphology, a low density such as 30 seeds/m^2^ influencing the production of bigger flowers, thus increasing the yield of polyphenols and terpenes production. Therefore, hemp inflorescences can be regarded as valuable by-products of fibre production, for their valorisation in the food and beverage industry in addition to cosmetics and perfumery.

## 1. Introduction

Industrial hemp is a *Cannabis sativa* L. chemotype grown for many hundreds of years in Europe and characterized by a low amount of the psychoactive cannabinoid delta-9-tetrahydrocannabinol (THC). During the Middle Ages, hemp was an important crop all around Europe, notably for the textile industry. Over the years, with the rise of the petrochemical industry, the cultivation of hemp has gradually been abandoned in favour of the cultivation of THC-rich *Cannabis* chemotypes. In 1995, a resolution of the Swiss Federal Offices of Public Health, Police and Agriculture authorized the cultivation of *Cannabis* within the country if its intended use was not as a drug [1]. In 1999, the European Union enacted an ordinance authorizing the cultivation of hemp with a THC content lower as 0.2% *w*/*w* for fibre and seed production [2]. In 2020, 71 different industrial hemp varieties are currently authorized for cultivation in the European Union [3].

Hemp is a profitable and multi-purpose crop, fitting into sustainable farming systems. The whole plant can be used in different kinds of applications: fibres from the straw are used in the textile and construction industries, as well as for energy production, and the oil and proteins extracted from the seeds are valuable compounds for the food and cosmetic industry because the seeds are rich in polyunsaturated fatty acids, in particular in gamma-linolenic acid and linoleic acid, in which the average Western diet is deficient [4]. Research on *Cannabis* phytochemicals has been mainly focused on cannabinoids and their pharmacological properties, and has been limited so far for various reasons, one of them being its classification as an illegal crop for many years. With the change in the regulations, low or non-THC *Cannabis* cultivars, also called hemp or fibre-type *Cannabis*, interest has grown in recent years. Moreover, inflorescences of fibre-type hemp continue to be an unexploited by-product of the fibre industry, while in addition to the cannabinoids, hemp is a rich source of phytochemicals such as terpenes and phenolics [5,6]. 

Phenolic compounds, and in particular flavonoids, are well known for their health-promoting effects, such as anti-oxidant, anti-inflammatory, anti-viral but also anti-cancer activities [7]. They are therefore seen as beneficial components of a healthy diet, as disease-preventing dietary compounds. The antioxidant property of flavonoids is also of interest in the cosmetic industry, since oxidative stress leads to skin aging [8].

Other compounds of interest produced by hemp are terpenes, which are responsible for its characteristic aroma. These volatile compounds, mainly mono- and sesquiterpenes, also possess beneficial biological activities, such as anti-inflammatory, anxiolytic and anti-depressant activities [9]. For example, the ability of beta-caryophyllene, one of the most predominant hemp sesquiterpenes, to bind with the Cannabinoid receptor type 2 (CB2), suggests a synergistic effect of the terpenes with the other hemp phytochemicals on health benefits [9]. 

Hemp sowing densities are known to influence its fibre and oil content [10,11,12]. A low seeding density of 30 plants/m^2^ is expected to favour the production of essential oils [13], a density of 150 plants/m^2^ is expected to favour the production of seeds and oil, while a density of 300 plants/m^2^ is favourable to the production of long fibres [14].

However, very little is known about the influence of the sowing density on the polyphenol content, nor about the evolution of the flavonoids and terpenes composition during the plant development in fibre-type hemp. In the present study, 8 different industrial hemp cultivars were grown outdoors in Wädenswil, Switzerland: the six monoecious cultivars Felina 32, Futura 75, Fedora 17, Fibror 79, Santhica 27 and Santhica 70, as well as the two dioicous cultivars KC Virtus and Finola. The goal of the present study was to understand the influence of growing conditions, harvest period and phenological stages on the plant chemical composition, focussing on total phenolic content and flavonoid composition, antioxidant activity, terpenes composition and on the morphological parameters of the plant. Therefore, the objective of this investigation was to gain insights into the optimal sowing density and optimal harvest time of different industrial hemp cultivars, in order to maximize the quantity of compounds of interest, and based on our results, to propose suitable future applications of the industrial hemp inflorescences in the field of food and cosmetics.

## 2. Results

### 2.1. Effect of Ripening and Sowing Density on the Plant Morphology

For each harvest date, 20 to 30 plants were collected, and the different parts weighed. The proportion of the inflorescence, the leaves and the stems are reported in Table S1.

The first comparison was made by studying the development of the proportion of the different parts of the plant overtime. In all species, except Finola, the proportion of the inflorescences increases with maturation, reaching their maximum in the last harvest in September (Table S1).

Fedora 17 and Finola show the highest proportion of inflorescences (representing more than 40% of the plant) while Santhica 27 and 70 show the smallest proportion of inflorescences (representing less than 25% of the plant) at their optimal ripeness stage.

**Figure 1:**
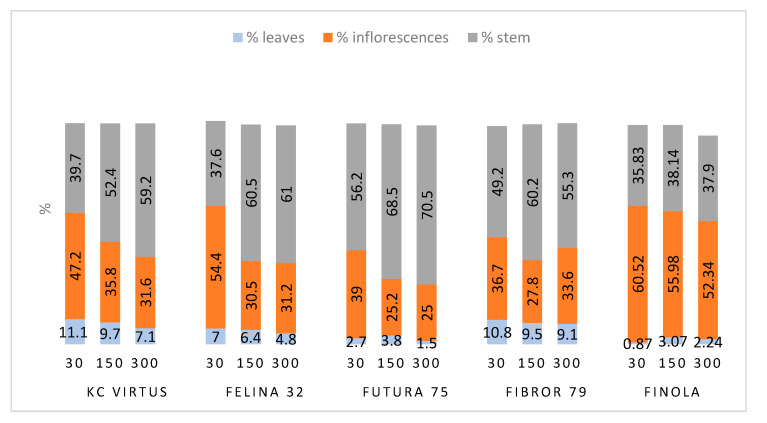
Proportions of leaves, inflorescences and stems of five hemp cultivars sown at 3 different sowing densities and harvested in September 2019 (end of August for Finola).

A low sowing density was shown to have an impact on the plant morphology. KC Virtus, Felina 32, Futura 75, Fibror 79 and Finola were planted at different densities. Plants sown at a density of 30 seeds/m^2^ and harvested at the end of September (full flowering stage for KC Virtus and Fibror 79; end of flowering stage for Felina 32, Futura 75 and Finola) developed bigger flowers than plants sown at densities 150 and 300 seeds/m^2^, as shown in Figure 1. Their stems also had a larger diameter (Table S1). With regard to the densities of 150 and 300 plants per m^2^, no significant morphological differences could be observed, the proportion of stems and flowers being comparable, as already described [15]. This can be explained by the fact that 150 and 300 plants/m^2^ are both considered a high planting density. The proximity of the plants causes a competition for access to light, which leads to a greater increase in stem length and inhibits growth in stem diameter [15], compared to a lower planting density (here 30 plants/m^2^).

### 2.2. Cannabinoid Acids Content of the Studied Cultivars

Cannabinoids are biosynthesised in plant tissues in the acid form. Cannabinoic acids such as cannabidiolic acid (CBDA) and cannabigerolic acid (CBGA) are the dominant cannabinoids constituents of fibre type *Cannabis sativa* [16]. Tetrahydrocannabinolic acid (THCA), CBDA and CBGA were the only forms detected in our samples. Each hemp sample was frozen on the same day within a few hours after the harvest, preventing the conversion of cannabinoid acids into their neutral forms via a decarboxylation reaction, which can be triggered by heat and light during the drying process [17]. As previously described [16], the fresh inflorescences of the present study were shown to contain moderate levels of cannabinoid acids CBDA and low levels of THCA, with the exception of cultivars Santhica 27 and 70, which were characterised by significantly lower levels of THCA and CBDA, but significantly higher level of their precursor CBGA in comparison with all other cultivar studied (*t*-Student test, Table 1).

All eight cultivars in the present study comply with European and Swiss regulations by containing less than 0.2% THC in its THCA form, the main psychoactive constituent of *Cannabis sativa*. They also comply with their commercial description, stating for all of them a content <0.2% THC, and 0% THC for Santhica 27 and 70.

Five different chemotypes of *Cannabis* are described in the literature, based on the THCA/CBDA ratio [18]. Finola, Felina 32, Fibror 79, Futura 75, KC Virtus (male and female) and Fedora 17 had a low THCA/CBDA ratio (<1), classifying them as chemotype III, typical for fibre-type plants. Santhica 27 and Santhica 70 were the only two cultivars where no THCA was detected and in which the CBDA levels were significantly lower than in other cultivars (<0.2 mg/g). Moreover, CBGA was detected in significantly higher concentrations compared to the other cultivars. This high CBGA content with undetectable THCA classifies Santhica 27 and 70 as chemotype IV.

### 2.3. Total Phenolic Content and Antioxidant Activity

The total phenolic content (TPC) and the antioxidant activity of the hemp samples collected between July and September were measured using spectrophotometric methods (Table 2).

The TPC ranges from 22.05 mg GAE/g *dw* (Fedora 17, density 150, harvested on the 22nd of July at early flowering stage), to 4.72 mg GAE/g *dw* (Santhica 70, density 300, harvested on the 10th of September at the end of flowering), and are in line with previous studies on industrial hemp species [19,20].

The TPC decreased significantly during the flower’s development for all cultivars studied, by a range of 25.5% to 76.3% between July and September, depending on cultivars. The highest TPC was found in the inflorescences harvested at early flowering stage in July for all cultivars and all sowing densities. This phenomenon has already been described for other fruits, such as peaches [21] or medlar fruits [22], but to our knowledge, never for hemp inflorescences. As described by Pourcel et al., polyphenolic compounds, such as flavonoids, can be oxidised within the plant due to an increased activity of different polyphenol oxidases and peroxidases during normal seed and plant development [23]. This oxidation process offers a physico-chemical protection of the plant and seeds tissues against different stresses [23].

The DPPH radical scavenging activity of all the extracts was also evaluated and decreases as well over the maturation period. The antioxidant activity, as determined by DPPH assays, had a significant correlation with the total phenolic content calculated for all flowering stages, sowing densities and cultivars (R^2^ = 0.96, Table S2). The extract of Fedora 17, harvested in July (early flowering; concentration 3.2 µg/mL), was able to scavenge 43% of DPPH radical and was therefore the most effective antioxidative extract. In comparison, the extract of Santhica 70, harvested in September (end of flowering), which contained 79% less total polyphenols, scavenged only 8% of the DPPH radical at the same concentration.

When comparing the different data obtained, Fedora 17 (density 150), Felina 32 (density 300), harvested in July and KC Virtus (density 30) harvested at the end of August (all in early flowering stage), were the richest in total polyphenols, showing the highest antioxidant activity, whereas Finola (density 150), Futura 75 (density 150) and Santhica 70 were the poorest. It should be noted that for the KC Virtus variety grown at a density of 150 plants per m^2^, the TPC values and antioxidant activities remain stable throughout the early flowering period, before dropping during full flowering.

### 2.4. Flavonoids Composition

HPLC-UV-MS/MS analysis revealed the presence of several flavonoids within the inflorescences of the different cultivars. In total, fifteen different compounds were identified based on retention time, absorbance spectra, exact mass and mass fragmentation patterns (Table 3). When possible, the identification was confirmed using the commercially available reference standard.

The identification of the glycosylated flavonoids was mainly realised based on MS and MS/MS data acquired in the positive ion mode. Structure elucidation of flavonoids glycosides using tandem mass spectrometry has been extensively described [24,25,26]. Important structural information can be obtained on the carbohydrate sequence, the aglycone part and on the *C*– or *O*– linkage between the aglycone and the glycoside. Fragmentation spectra of each compound are accessible in the supplementary material (Figures S1–S9).

However, for compounds **3** and **4**, the observation of the MS/MS fragmentation patterns recorded using high collision energy (70 eV) did not allow a distinction to be made between kaempferol or luteolin moieties. The absorbance spectra of the two compounds were hence recorded and compared (Figures S10 and S11). Shifts can be observed in the maximum absorbance wavelengths between kaempferol (max. at 265 nm and 365 nm) and luteolin (max. at 252 nm, 267 nm and 349 nm) (Table S3 and Figures S10 and S11). Compounds **3** and **4** showed similar absorbance spectra to the one of luteolin (max. absorbance at 255 nm, 267 nm and 350 nm), and were therefore identified as luteolin-*C*-glucoside (**3**) and luteolin-*C*-hexoside-*O*-rhamnoside (**4**).

The flavonoid content varied from 7.37 mg/g of dry weight (Felina 32, density 300, harvested on the 22nd of July, early flowering stage) to 0.06 mg/g (Santhica 70, density 300, harvested on the 10th of September, end of flowering stage) (Tables S3–S12). As already observed for the total phenolic content and the antioxidant activity (Table 2), the content in flavonoids decreased with flower maturation, and was higher in the inflorescences harvested in the early flowering stage, for all cultivars.

Regarding the composition, even if every cultivar differed in the concentration of the flavonoid contents detected, the female inflorescences contained mainly flavone derivatives, apigenin, luteolin and chrysoeriol glycosides being the most abundant compounds, regardless of the cultivar. Luteolin-7-*O*-glucuronide (**8**) was the most abundant flavonoid in all cultivars, followed by apigenin-7-*O*-glucuronide (**9**), vitexin-2’’-*O*-glucoside (**6**) and vitexin-2’’-*O*-rhamnoside (**7**), which concurs with the findings of Vanhoenacker et al. [27] 

Male inflorescences of the two dioicous cultivars of the study (KC Virtus and Finola) contained overall less flavonoids than female inflorescences, as well as two unique flavonol compounds, quercetin-*O*-sophoroside (**1**) and kaempferol-*O*-sophoroside (**5**) (Tables S6–S8 and S13), found for the first time by Ross et al. in the pollen of *Cannabis sativa* [28].

**Figure 2:**
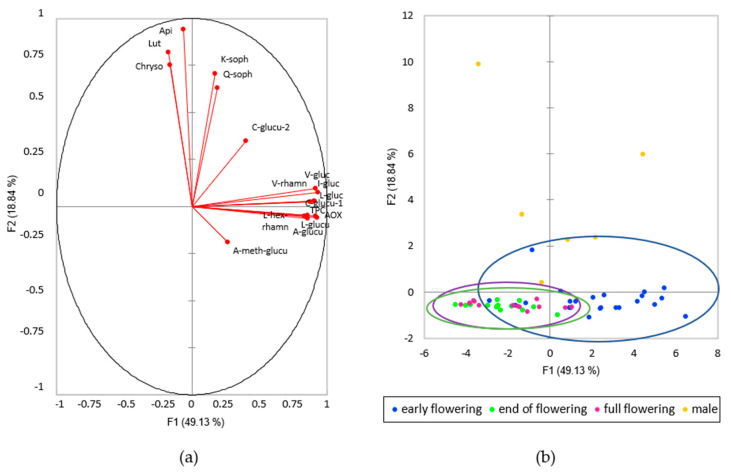
Principal Component Analysis (PCA) of the flavonoid profile, total phenolic content and antioxidant activity of the different hemp cultivars; (**a**) direction of each of the compounds analysed on the first two principal components; (**b**) biplot of the first two principal components of PCA. Colours represent the phenological stage.

The data obtained on the total phenolic content, the antioxidant activity and the concentration of the different flavonoids within the hemp extracts were statistically analysed using the Principal Component Analysis (PCA) method. The first outcome of the analysis is given by the biplot in Figure 2 (Figure 2a represents the direction of each variable that went into the PCA; Figure 2b is the biplot of the first two principal components of PCA). The total seventeen principle components explain 100% of the total variance. The two first principal components, PC1 and PC2, represent 68% of the variance. In PC1 the corresponding loading factors were positive for all flavonoid glycosides, and negative for the aglycone parts (Figure 2a). For greater ease of comprehension, the data were coloured by their phenological stage.

Hemp samples of all cultivars harvested in early flowering stage (circled in blue), full flowering (circled in violet) and end of flowering stages (circled in green) are clearly separated along the PC1 axis. Early flowering samples showed higher content in different flavonoids, in total phenolic content and antioxidant activity than inflorescences collected at full flowering or end of flowering stages. Inflorescences at full flowering and end of flowering group together, indicating a similar content in terms of flavonoids, total phenolic content and antioxidant activity (Figure 2; Table 2; Figures S4–S13).

**Figure 3:**
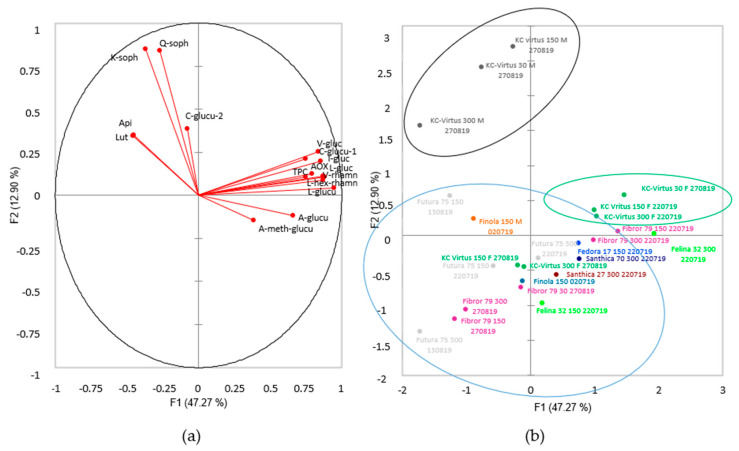
PCA of the flavonoid profile, total phenolic content and antioxidant activity of the different hemp cultivars; (**a**) direction of each of the compounds analysed on the first two principal components; (**b**) biplot of the first two principal components of PCA. Colours represent samples from the same cultivar.

To analyse the effect of the sowing density on the flavonoid content of the different cultivar, a second PCA was conducted, this time by keeping only the data from the harvest date showing the highest total phenolic and flavonoids content, and antioxidant activity, for each cultivar. The two first components account for 60.2% of the variance. The data presented in Figure 3 were coloured according to cultivars.

The result of the second PCA (Figure 3) shows that the effect of the sowing density differs depending on the cultivars.

For the cultivar Felina 32, it can be seen in Figure 3b (light green colour) that the two densities are apart in the biplot graph, indicating a difference in chemical composition between the two planting densities. Looking in detail, it can be seen that cultivar Felina 32 grown at the density of 300 seeds/m^2^ contains significantly more flavonoids than the inflorescences from density 150 (Table S5; *p* < 0.01; +40%). For the cultivars KC Virtus, Fibror 79 and Futura 75, no differences can be seen between the densities 150 and 300 seeds/m^2^ harvested at the same phenological stage, as shown in the blue circle in Figure 3b. Looking in detail at the flavonoid composition, the TPC and the antioxidant activity, no significant differences are seen. However, the cultivar KC Virtus sown at density 30 seeds/m^2^ and harvested at the early flowering stage (end of August 2019) grouped with the two samples of densities 150 and 300 harvested at early flowering stage in July (Figure 3b; green circle). Looking in detail, this sample and the one harvested in full flowering stage in September contained significantly more total polyphenol and flavonoids than the inflorescences harvested at the same period and sown at a higher sowing density, even though they were at the same phenological stage (Table 2; Tables S6–S8). The plants sown at 30 seeds/m^2^ also showed differences in terms of plant morphologies, with bigger inflorescences (Figure 1). The same observation was made for the cultivar Fibror 79, where the inflorescences of the plants grown at density 30 seeds/m^2^ contained significantly more total polyphenols and flavonoids on the 27th of August (end of early flowering stage) than inflorescences of the densities 150 and 300, yet at the same flowering stage (Table 2; Table S9). Even though no trend has emerged, however, it appears that for some cultivars a low planting density would have a positive impact on the concentration of polyphenols within the flowers. 

Male plants of the dioicous cultivars KC Virtus form a cluster, as can be seen in Figure 3b (circled in black). This is explained by the fact that male inflorescences of KC Virtus contain two unique flavonoids, kaempferol-*O*-sophoroside and quercetin-*O*-sophoroside [28], as well as 2 aglycones, apigenin and luteolin (arrows on Figure 3a), but also contain overall fewer amounts of flavonoids and total phenolic compounds, having a smaller antioxidant activity than female inflorescences (Table 2; Tables S6–S8). The male inflorescences of Finola cultivar contain less kaempferol-*O*-sophoroside and quercetin-*O*-sophoroside, and do not contain any aglycones (Table S13), and therefore are not clustered with KC Virtus male inflorescences.

As the antioxidant activity of a plant extract depends on the content of various class of phytocomponents, a correlation analysis was performed (Table S2). This analysis revealed that several specific flavonoid compounds of hemp inflorescences were positively correlated to the antioxidant activity: luteolin-7-*O*-glucuronide and luteolin-*C*-hexoside-*O*-rhamnoside having the highest degree of correlation (0.81, *p* < 0.05), followed by isoorientin-2’’-*O*-glucoside (r = 0.76, *p* < 0.05), luteolin-*C*-glucoside (0.72, *p* < 0.05) and chrysoeriol-*O*-glucuronide isomer 1 (0.71, *p* < 0.05). Those compounds are therefore regarded as the most potent antioxidative compounds among all flavonoids found in hemp inflorescences. On the other hand, the three aglycones—luteolin, apigenin and chrysoeriol—found in few samples were negatively correlated with the antioxidant activity.

The overall analysis reveals that cultivars Felina 32, grown at 300 seeds/m^2^, KC Virtus, grown at 150 and 300 seeds/m^2^, Fibror 79, grown at 150 or 300 seeds/m^2^ and KC Virtus, grown at 30 seeds/m^2^ (all harvested at the early flowering stage), contain the highest amount of flavonoids, of total polyphenols as well as have the highest antioxidant activity. As the consumption of dietary flavonoids, and among them of flavonols and flavones, have been shown to have protective effects against ovarian and colorectal cancers, and to prevent coronary heart disease [29], those three cultivars are therefore presumed to be the most potent in terms of health benefit.

### 2.5. Terpenes Composition

Terpenes are the major constituents of the essential oils of *Cannabis sativa* and are responsible for its distinctive flavour and aroma. Twenty-nine terpenes were identified within the extracts of the hemp inflorescences harvested from July to September by means of GC-MS and comparison with libraries, and confirmed when possible using the commercially available reference standards (Table S14). The fourteen most abundant terpenes were quantified by mean of gas chromatography coupled to flame ionisation detector (GC-FID; Table S15).

In general, all the hemp samples analysed in this study displayed the same qualitative profile, whereas the quantitative profiles differed depending on cultivars (Table S15). Two sesquiterpenes, beta-caryophyllene and alpha-humulene, as well as 3 monoterpenes, myrcene, alpha-pinene and beta-pinene, were the most abundant in all cultivars, in line with previous publications on industrial hemp [30,31,32].

The first observation is that the terpene content changed during the development of the inflorescence. The terpene content increased throughout the early flowering stage until the full flowering stage, then decrease at the end of flowering.

**Figure 4:**
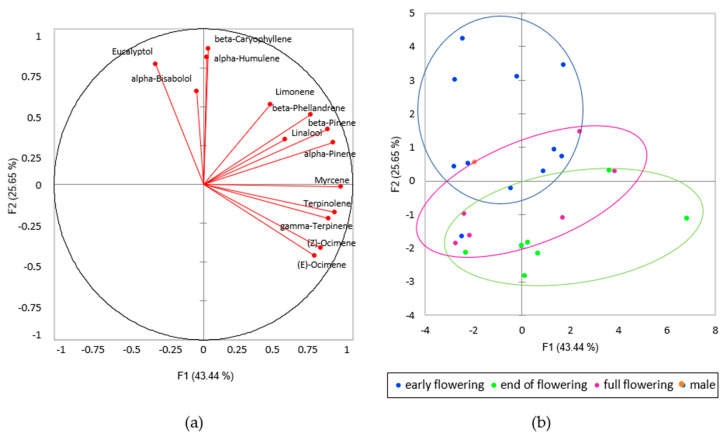
PCA of the terpene profile of the different hemp cultivars; (**a**) direction of each analysed compound on the first two principal components; (**b**) biplot of the first two principal components of PCA. Colours represent the phenological stage.

The data obtained on the concentration of the different terpenes within the hemp extracts were analysed using the PCA method. The two first components account for 69% of the variance. The first outcome is given by the biplot in Figure 4, where data were coloured by their phenological stage. Figure 4b show that the proportion of monoterpenes and sesquiterpenes changed during the season, and that three different clusters can be identified. Early flowering samples form a first cluster (circled in blue in Figure 4b) where samples are characterised by a higher content in all three main sesquiterpenes and eucalyptol (direction of arrows in Figure 4a), sesquiterpenes representing more than 70% of the total terpenes, giving a monoterpene/sesquiterpene ratio ≤ 1.

With flower development, the sesquiterpene content progressively decreased, and monoterpenes such as myrcene, alpha- and beta-pinene, terpinolene or linalool increased, as seen in full flowering (violet cluster) and end of flowering (green cluster) clusters in Figure 4. In older flowers harvested late in the season, the monoterpenes represented more than 50% of the total terpenes. Santhica 27 was the only cultivar where sesquiterpenes remained predominant. Most of the studies on hemp aromas conducted to date state that monoterpenes predominate over sesquiterpenes, except for the study of Vuerich et al. [33], where a higher proportion of sesquiterpenes is reported. In the study of Vuerich and collaborators, flowers were cut well before the harvest in order to allow the plants to issue new inflorescences a second time. Until now, no studies have described the development of the terpene composition of hemp; however, our study would suggest that a harvest of the hemp inflorescences in the early flowering stage can produce an aromatic profile richer in sesquiterpenes.

**Figure 5:**
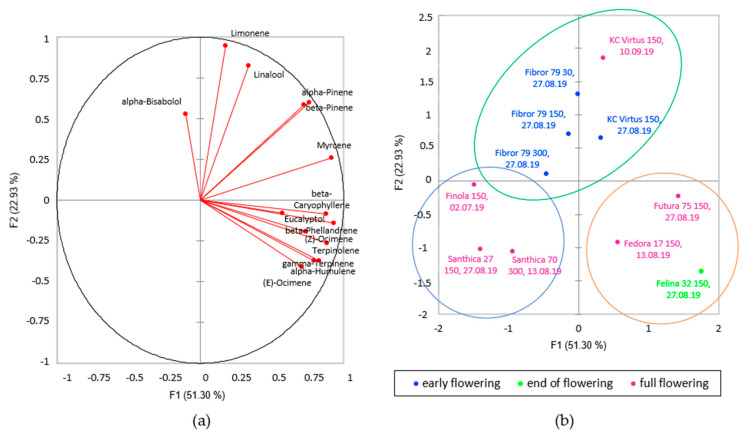
PCA of the terpene profile of the different hemp cultivars; (**a**) direction of each analysed compound on the first two principal components; (**b**) biplot of the first two principal components of PCA. Colours represent the phenological stage.

Thus, in order to observe aroma differences between the cultivars, the samples harvested late in the season close to full flowering, at full flowering or at end of flowering stages were used to compare the different cultivars and to look for aroma similarities. A second PCA was realized with the data obtained by the means of GC-FID. The two first components explain 74.23% of the variance as can be seen in Figure 5. The analysis revealed similarities in the terpene composition of different cultivars, which are summarised in Table 4. Three different clusters are formed along PC1 and PC2 axes. It was possible to identify three different aromatic profiles among the different cultivars.

The first cluster (circled in blue in Figure 5b) formed by Santhica 27 and 70 and Finola (female flowers) harvested at full flowering stage, contain low amounts of all terpenes compared to the other cultivar (268.4 ± 40.4 mg/kg). It should be noted that the Finola cultivar was characterised by the presence of relatively high amounts of (*E*)-ocimene, as already described in other studies [34].

The second cluster is formed by Felina 32 harvested after full flowering, Fedora 17 and Futura 75 samples harvested at full flowering stage (circled in orange in Figure 5b). Those cultivars contained the highest total terpene content among all other cultivars (820.7 ± 171.9 mg/kg) and produced the highest concentrations of monoterpenes over sesquiterpenes (average mono-/sesquiterpenes ratio of 2.3). It is interesting to note that although the content in phytochemicals of outdoor cultivated hemp is highly dependent on environmental and climatic conditions, the percentage of the main terpene compounds in Felina 32 and Futura 75 are in line with previously published data [30,31]. Those 3 cultivars are characterised by a high amount of myrcene, terpinolene, beta-phellandrene and (*E*)-ocimene (Figure 5a). 

The third cluster is formed by KC Virtus (female flowers) and Fibror 79 cultivars harvested end of August just before full flowering and at full flowering stages (circled in green in Figure 5b). Those two cultivars contained two times more monoterpenes than sesquiterpenes and were characterised by a high amount of limonene, linalool and bisabolol (Figure 5a).

Within the third cluster, the influence of the sowing density can be observed. The three sowing densities of Fibror 79 harvested at the same phenological stage just before full flowering, were analysed. Fibror 79 sown at 30 seeds/m^2^ produced a higher quantity of terpene than densities 150 and 300 (Table S15), which is in line with a previous publication [13]. When taking also into account the KC Virtus cultivar sown at 150 and 300 seeds/m^2^, it can be observed that the proportion as well as the quantity of sesquiterpenes increased with increasing sowing density. In Fibror 79 and KC Virtus, the quantities of beta-caryophyllene, alpha-humulene and alpha bisabolol increased with higher sowing density (Table S15). Therefore, increasing the sowing density might induce the production of more sesquiterpenes within the inflorescences of some fibre-hemp cultivars, whereas decreasing the sowing density might increase the overall terpene content.

Regarding the low abundance compounds, the development of their quantity according to the density of plantation is cultivar dependent. In Fibror 79, limonene, alpha and beta-pinene decreased with increasing sowing density. Interestingly, (E)-ocimene and terpinolene both increased with increasing sowing density. In KC Virtus cultivar, the increase was observed for eucalyptol and linalool (Table S15). Low abundance terpenes of hemp are thought to be responsible for aroma differences between cultivars and may have been the aromatic cues identified by growers when it comes to drug-type hemp [9].

Finally, the terpene composition of male and female inflorescences of cultivar KC Virtus was compared. Except for the highest quantity of terpenes produced by female flowers, the male inflorescences contain in proportion more sesquiterpenes, and notably beta-caryophyllene and alpha-humulene as well as more myrcene. Female flowers on the other hand, contain more alpha and beta-pinene, limonene and terpinolene (Table S15).

## 3. Discussion

The use of fibre-type hemp inflorescences, which continues to be an under-utilised waste product of the fibre industry [30,31], has been little studied, even though these inflorescences are a valuable part of industrial hemp, rich in antioxidant and aroma compounds. 

In order to enhance the value of the inflorescences of industrial hemp varieties, our study provides a comparison of different cultivars in terms of antioxidant and aroma compounds, and provides scientific support and impetus to optimise the cultivation and harvesting parameters to obtain a quality of hemp tailored to the desired industrial application. 

**Figure 6:**
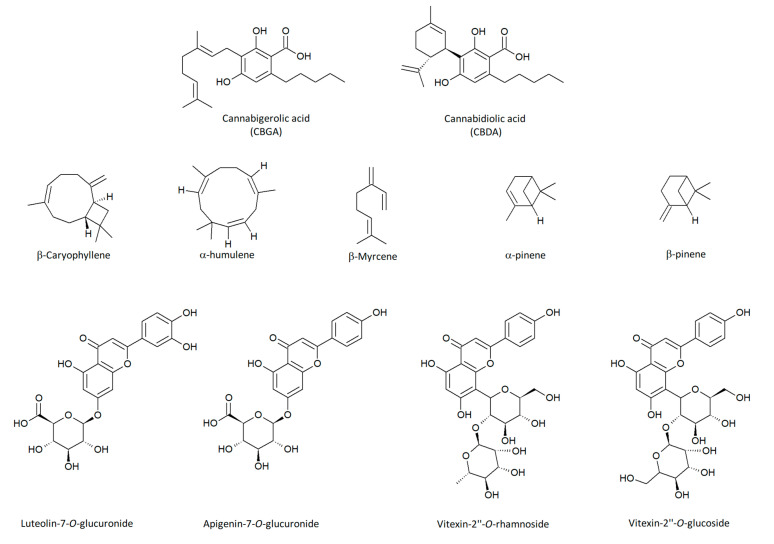
Chemical structures of the main compounds detected in fibre-type hemp cultivars.

Fifteen different flavonoids were identified in the inflorescences of the hemp cultivars studied. The main compound was luteolin-7-*O*-glucuronide, followed by apigenin-7-*O*-glucuronide, vitexin-2’’-*O*-glucoside and vitexin-2’’-O-rhamnoside (Figure 6). Two unique flavanol glycosides were also detected in male flowers of the two dioicous cultivars: quercetin-*O*-sophoroside and kaempferol-*O*-sophoroside. 

The terpene profile of all hemp cultivars was dominated by two sesquiterpenes, beta-caryophyllene and alpha-humulene, and three monoterpenes, myrcene, alpha-pinene and beta-pinene (Figure 6).

Our study shows that the primary factor which influences the polyphenol and flavonoid content, the antioxidant activity, as well as the terpene content, is the phenological stage of the plant, coupled with the harvest date. We found that the total polyphenol and flavonoid content of all hemp cultivars decreases with plant maturation. This has already been observed in different cultivated fruits [21,35], but is described here for the first time with this level of detail and so many data points for the inflorescences of several different fibre-type hemp cultivars. The antioxidant activity is strongly correlated to the content in total polyphenols, and specifically to the content of luteolin-7-*O*-glucuronide and luteolin-*C*-hexoside-*O*-rhamnoside. Therefore, the antioxidant activity of the extracts was higher when the inflorescences were harvested in early flowering stage.

In order to produce extracts with a higher content of polyphenols and flavonoids, which have the highest antioxidant activity, it is therefore recommended to harvest the hemp inflorescences at their early flowering stage, before full flowering. Polyphenols, including flavonoids, are widely consumed by humans as part of a balanced diet [36]. They are found in edible plants, but also in beverages such as tea or coffee [37]. Through their antioxidant activity, polyphenols and flavonoids play a role in the prevention of various diseases associated with oxidative stress and are therefore beneficial for human health [38]. The hemp content in flavanol glycosides, found only in male plants, reach 39.9 mg/100 g dry weight of hemp, therefore being in the same range as the richest dietary sources such as leek and broccoli [37]. Flavones, which are much less common than flavanols in fruits and vegetables [37], are major constituents of the hemp flowers extracts. The content of flavones found in the cultivars studied was higher than the one found in celery, one of the most important edible sources of flavones identified by Manach et al. [37]. Therefore, hemp inflorescences can be an interesting source of edible polyphenols for the food and wellness industries.

Our study established that Felina 32, KC Virtus and Fibror 79 were the highest producers of polyphenols and flavonoids, of all 8 cultivars studied, when harvested in at early flowering stage. KC Virtus has the advantage of being a dioicous cultivar, therefore also allowing the harvest of male plants producing flavanol glycosides.

Terpenes, which are responsible for the characteristic odour and flavour of *Cannabis sativa*, were shown in our study to increase with the development of the hemp inflorescence. The terpene content increases from July to the end of August, and the proportion of monoterpenes and sesquiterpenes varies from early flowering to full flowering and end of flowering stages. Therefore, the optimal harvest time depends on the desired application. For example, the hemp cultivars harvested early in the season in order to maximise their polyphenol content, and therefore their antioxidant activities, will have a terpene profile dominated by sesquiterpenes. The scent will consequently be described as spicy, hoppy and woody [9]. Moreover, sesquiterpenes such as beta-caryophyllene and alpha-humulene possess biological activities such as anti-inflammatory [39] and gastroprotective properties [9], which together with the antioxidant activity of polyphenols, could be of interest for the development of new functional food and beverages as well as cosmetics. Those two sesquiterpenes have also been shown to express anti-depressant and anxiolytic activities [40] of interest for the health and wellness-being industry. 

With flower development, the monoterpenes increase and then exceed the sesquiterpenes, therefore changing the aromatic profile toward a more citric and fresher scent. Low abundance terpenes also build the characteristic aroma of each hemp cultivar [9]. When harvested after full flowering, Fedora 17 and Finola were characterised by a higher amount of (Z) and (E)-Ocimene (citrus, tropical), Felina 32 and Futura 75 by beta-phellandrene and terpinolene (citrus, pepper, pine). KC Virtus harvested at the full flowering stage is characterised by a higher eucalyptol content. 

Even if the polyphenol content and antioxidant activity are no longer of interest when the inflorescences are harvested at later date, other aroma applications can be considered. One of the most interesting could be to use the aroma similarities found between hops and hemp, both belonging to the *Cannabaceae* family, in beer brewing. Principal aroma constituents of hops are myrcene, beta-caryophyllene and humulene, together with smaller amounts of linalool and geraniol (floral aroma) and limonene and alpha-terpineol (citrus aroma) [41,42]. As we found out during this study, certain fibre-type hemp cultivars, such as KC Virtus or Felina 32, contain those same aroma compounds in high amounts, and could therefore be used to replace hops for dry-hopping in beer-making, hop cultivation being likely to suffer the effects of global warming in the years to come [43,44]. However, further analysis of the aroma and taste-active constituents using sensory-guided analytical techniques are required.

Regarding the morphological parameters of the hemp plants that were monitored during this study, our findings are that the flower proportion in early flowering stage represent less than 20% for most cultivars, except for Fedora 17 (25%) and Finola (61%). The flowers’ size increased with maturation, except for Finola.

A decrease in sowing density allowed the hemp to grow bigger flowers, therefore increasing the production yields of both antioxidant and aroma compounds compared to higher sowing densities. Decreasing the sowing density to around 30 seeds/m^2^ can also influence the production of terpenes, toward more sesquiterpenes or more monoterpenes, depending on cultivars, as it was observed with Fibror 79 and KC Virtus.

Therefore, the hemp inflorescences of the 8 different cultivars studied, which can all be by-products of hemp fibres or oil industry, are valuable sources of antioxidant and aroma compounds. They can be valorised in diverse fields, for the development of food and beverages with health benefits, in the flavouring sector with a focus on hops alternative products, as well as in the cosmetics industry.

## 4. Materials and Methods 

### 4.1. Chemicals and Reagents

The flavonoids aglycones luteolin, chrysoeriol and kaempferol were obtained from Extrasynthèse S.A. (Lyon, France). Vitexin-2-O-rhamnoside, apigenin-7-*O*-glucuronide, apigenin, quercetin, cannabigerolic acid (CBGA), tetrahydrocannabinolic acid (THCA) and cannabidiolic acid (CBDA) were purchased from Sigma-Aldrich Chemie GmbH (Buchs, Switzerland) and luteolin-7-*O*-glucuronide from HWI-group (Rülzheim, Germany). (+)-alpha-Pinene, (−)-beta-Pinene, beta-Myrcene, Ocimene, L-Linalool, (E)-beta-Caryophyllene, alpha-Humulene, (−)-alpha-Bisabolol, S(−)-Limonene, alpha-Phellandrene, 1.8-Cineol, Terpinolene and gamma-Terpinene were purchased from Sigma-Aldrich Chemie GmbH (Buchs, Switzerland).

Acetonitrile (LC-MS grade) was supplied by VWR International GmbH (Dietikon, Switzerland). Water (LC-MS grade) and methanol (HPLC grade) were supplied by Carl Roth AG (Arlesheim, Switzerland). Trifluoroacetic acid (TFA) and dimethysulfoxide (DMSO) were supplied by Sigma-Aldrich Chemie GmbH (Buchs, Switzerland).

### 4.2. Plant Material and Agricultural Conditions

Eight industrial hemp cultivars of *Cannabis sativa* L. (Finola, Fedora 17, KC Virtus, Santhica 27, Santhica 70, Fibror 79, Felina 32, Futura 75) were cultivated in the garden of the Grüental campus of the Zurich University of Applied Sciences (ZHAW) in 2019 (Wädenswil, Switzerland, latitude 47°13′02.3″ N, longitude 8°40′57.5″ E). The experiment lasted from 7th of May 2019 (sowing) until 25th of September 2019 (uprooting). The main characteristics of the cultivars are reported in Table 5.

The cultivars of the study are in compliance with the EC regulation (No 809/2014 of 17 July 2014) and are therefore authorised for cultivation in Switzerland.

The soil is a deep sandy clay soil. The mineral nitrogen content was measured in the soil at various depths before fertilisation. The layer between 0 and 30 cm deep contained 22 kg N–NO3/ha, the layer between 30 and 60 cm contained 8 kg N–NO_3_/ha and the layer between 60 and 90 cm contained 2 kg N–NO3/ha. Fertilisation using Plantaktiv Typ K (16% N, 6% P_2_O_5_, 26% K_2_O, 3.4% MgO; Hauert, Grossaffoltern, Switzerland) was done on 07.06.2019 and 26.06.2019. After solubilisation in water, the fertiliser was distributed on the field with an irrigation sprinkler (approx. 20 kg N/ha 4 weeks after sowing, approx. 40 kg N/ha 7 weeks after sowing). No pesticides were used, as well as no irrigation, except the water brought during the fertilisation. Some climatic parameters of the study site are given in Table 6.

Hemp seeds were sown by hand at a sowing depth of 4 cm and at different sowing densities (30/150/300 seeds/m^2^, depending on the species, as indicated in Table 1) on the 7th May 2019, in 7 rows with 1.05 m width, and 22 cm within rows. The lengths of the plots differed according to the sowing density: 20 linear metres were sown for the sowing density of 30 seeds/m^2^, 12 linear metres for the sowing density of 150 seeds/m^2^ and 8 linear metres for the sowing density of 300 seeds/m^2^. The borders of the strip were planted with the cultivar KC Virtus, in order to prevent an edge effect. These border plants were not harvested.

### 4.3. Harvests

Plants were harvested at four different periods, from early flowering to end of flowering in July 2019 (11 weeks after sowing), August 2019 (14 and 16 weeks after sowing) and September 2019 (18 weeks after planting). The phenological stage of each cultivar for each harvest date is indicated in Table 7 [45].

For the 300 and 150 plants/m^2^ densities, 1 m^2^ was harvested at each time point. For the 30 plants/m^2^ density, between 10 and 20 stems were harvested. Inflorescences, leaves and stems were separated. Half of the harvest was dried in a heating cabinet at 45 °C for 4 days, acclimatised at room temperature before the different part were weighed to determine the proportions of inflorescences, leaves and stems described in Figure 1. For the second half of the harvest, only the fresh inflorescences were kept and frozen at −20 °C immediately after the harvest, then crushed a couple of days after with liquid nitrogen and ground using an Ika A11 basic analytical mill. The ground samples were kept at −80 °C until further analysis (total phenolic content, antioxidant activity, cannabinoid acids, flavonoids and terpenes analysis).

### 4.4. Hemp Extraction for TPC, Antioxidant Activity and HPLC-MS/MS Analysis

In total, 56 hemp samples were analysed. A total of 30 g of each frozen sample was freeze-dried. Then, 0.4 g of dried powder was weighed in a 15 mL centrifuge tube and extracted with 5 mL of a 70:30 methanol:water mixture. The tube was sonicated in an ultrasound bath for 10 min, then centrifuged at 4000 rpm for 2 min. Extractions were made in duplicate.

The supernatant was filtered through 0.2 µm polytetrafluoroethylene (PTFE) filters (Chromafil extra, Machery-Nagel AG, Oensingen, Switzerland) into HPLC 2 mL amber vials and analysed directly through HPLC-MS/MS.

The remaining supernatant was used to determine total phenolic content and antioxidant activity.

### 4.5. Total Phenolic Content

The total phenolic content (TPC) of the extracts were determined by the Folin–Ciocalteu reagent method [46]. To obtain a working solution, 2 M Folin–Ciocalteu reagent was diluted in a ratio of 1:3 with water. Hemp extract was diluted 50 times so that finally 1 mL of the diluted extract was mixed with 1 mL of Folin–Ciocalteu reagent, 2 mL of a 20% anhydrous sodium carbonate solution, 2 mL of water, mixed well and left for reaction in the dark for 2 h. The absorbance of the reaction mixture was measured at 750 nm using a spectrophotometer (Genesys™ 10S, Thermo Fisher Scientific, Reinach, Switzerland). All samples were carried out in triplicate for each sample (mean ± SD). Gallic acid was used as a calibration standard and results were expressed as milligrams of gallic acid equivalent per gram of dry hemp (mg GAE/g). The calibration curve of gallic acid was in the calibration range of 0.005–0.05 mg/mL with a linear regression line of y = 20.351x − 0.0059 with R^2^ = 0.9962.

### 4.6. Antioxidant Activity

The antioxidant activity of the extracts was determined using DPPH assay following the protocol described by Pedan et al. [47]. The DPPH working solution was prepared by dissolving 24 mg of DPPH in 550 mL methanol and left to react in the dark for 24 h at room temperature to obtain an absorptance of 1.1 ± 0.2 units at 515 nm. The extract was then diluted 25 times in water, and 150 µL of the diluted extract was allowed to react with 2850 µL of the DPPH working solution. The absorbance at 515 nm was noted after an incubation time of 30 min using a spectrophotometer (Genesys™ 10S, Thermo Fisher Scientific, Reinach, Switzerland). The inhibition percentage for scavenging DPPH radical was calculated according to the equation:

$$\%\;DPPH\;radical\;scavenging\;activity=\;\left(\frac{ABSc-ABSs}{ABSc}\right)\;\times \;100$$

where ABS_c_ is the absorbance of the blank solution at 515 nm, and ABS_s_ is the absorbance of the sample after 30 min of incubation with DPPH at 515 nm.

### 4.7. HPLC-MS/MS Analysis of Flavonoids

LC-MS/MS analyses were conducted on a system consisting of an Agilent 1290 Infinity II chromatographic system coupled to an Agilent 6530 Q-TOF mass spectrophotometer. Separation of analytes was performed using an Agilent Poroshell 120 EC-C18 (2.1 × 150 mm, 2.7 µm) column preceded by a guard column (Agilent EC-18, 2.1 × 5 mm, 2.7 µm). The flow rate was set to 0.12 mL/min, and the column temperature set at 50 °C. The two elution mobile phases were made up of water +0.1% trifluoroacetic acid (TFA) (mobile phase A) and 95:5 acetonitrile: water +0.1% TFA (mobile phase B). Gradient elution was as follows: 0–1 min, 10% B; 3 min, 12% B; 4 min, 15% B; 18 min, 16% B; 20 min, 19% B; 21 min, 50% B; 22 min, 60% B, 24–33 min, 65% B; 34–38 min, 100% B. Re-equilibration time was 11 min. Injection volume was 1 µL. All samples were analysed in duplicate.

The MS analyses were performed using Agilent 6530 Q-TOF instrument in positive ionisation mode (ESI), in the spectral range of 100–1700 Da. Nitrogen served as the nebuliser and collision gas. The MS parameters were as follows: gas temperature, 320 °C; drying gas, 10 L/min; nebuliser, 50 psi; sheath gas temperature, 350 °C; sheath gas flow, 12 L/min; capillary voltage, 3500 V. For MS/MS analysis, four different collision energy were applied: 15/30/45/70 eV using Agilent auto MS/MS mode.

For the quantification, the reference standards were used when possible. When not, the respective aglycone was used as reference compound. Calibration curves were built using the peak area at the corresponding wavelength for each reference under the chromatographic conditions described above, and calibration solutions were analysed in duplicate. The equations of the obtained linear curves are available in Table 8.

### 4.8. Plant Extraction for GC-MS and GC-FID Analysis of Terpenes

In order to analyse the terpene profiles, an extraction approach was developed which enables a targeted extraction of the hemp mono- and sesquiterpenes directly out of the plant material, resulting in extracts with relatively low amounts of the co-extracted lipid fraction. For this purpose, 2 g of frozen hemp samples were extracted with 20 mL of acetonitrile, homogenised using a Polytron homogeniser at 1000 U/min for 1 min and further extracted under magnetic stirring for 30 min. After the extraction, the solids were allowed to sediment. Subsequently, 7 mL of the supernatant were removed and spiked with 70 µL of injection standard stock solution (amylacetate and methylpelargonat at 1 g/L). In the next step, 3 mL ultra-pure water were added to spiked supernatant and the preparation was vortexed for 30 s with 0.4 g of silica gel C18, in order to bind the co-extracted long chain fatty acids and other nonpolar compounds. After the sedimentation of the silica gel C18, 7 mL of the supernatant were removed and diluted with 20 mL ultrapure water, after which 4 g of sodium chloride were added and dissolved by shaking. Furthermore, 5 mL of hexane were added, and a liquid/liquid extraction was performed by shaking vigorously for 30 s. After phase separation, approximately 1 mL of organic phase was filled into a GC vial. The extracts obtained were used for both GC-MS screening and the quantitation by means of GC-FID.

### 4.9. GC-MS Analysis of Terpenes 

For compound identification, mass spectra were generated by TSQ 8000 Evo mass spectrometer coupled to a Thermo Trace GC equipped with a TriPlus RSH Autosampler (Thermo Scientific). The samples (1 µL) were injected on column. The separation of the volatiles was performed on a DB-FFAP capillary column (30 m × 0.25 mm, 0.25 µm film thickness; Agilent) connected with a fused silica pre-column capillary (5 m × 0.53 mm; Handel Müller GmbH) using helium as the carrier gas at a constant flow rate of 2.5 mL/min, by application of the following temperature program: 40 °C for 6 min, then raised to 240 °C at a rate of 7 °C/min and held at 240 °C for 25 min. The injection port was set at 50 °C, and the transfer line to 240 °C with an increase of 14.5 °C/s.

The identification was done in the electron ionization (EI) mode (70 eV). The spectra for identification were recorded in the full scan mode from 35–250 amu with a scan time of 11 ms. The ion source temperature was 220 °C. NIST mass spectral library software (version 2.2; Standard Reference Data Program of the National Institute of Standards and Technology, distributed by Agilent Technologies) was used to assist in compound identification. Most of the compounds identified by the MS-Spectra were verified by commercially available standards (see supporting information S14).

### 4.10. GC-FID Quantification of Terpenes

For the quantitation of the analytes identified by GC-MS, the experiments were performed using a GC system (Thermo Trace GC Ultra) equipped with a Flame Ionisation Detector (FID). Separation of the compounds was conducted on a column DB-1701 30 m × 0.25 mm (0.25 µm film thickness; Agilent Technologies, Basel, Switzerland), using helium as the carrier gas. The injection volume was 1 µL, and the flow rate 2.5 mL/min. The injector temperature was 200 °C with a split ratio of 1:10 and the FID temperature was 250 °C. The oven temperature was programmed as follows: the column was held initially at 40 °C for 5 min, then increased to 190 °C at 7 °C/min, then increased to 280 °C at 50 °C/min. For the GC-FID quantitation, standards and blanks were prepared. For blank, 7 mL of acetonitrile +70 µL of stock solution of injection standard at 1 g/L, then prepare as described above.

For calibration, three different concentrations of each analyte were prepared by diluting 30, 70 and 140 µL of the standard stock solution (1 g/L) in 7 mL of acetonitrile and 70 µL of stock solution of injection standard (1 g/L), then prepared as described above. The determined calculations showed a calibration range from 0.1 to 92 mg/kg and a coefficient of determination (R^2^) ≥ 0.999.

### 4.11. Statistical Analysis

Data analysis was done using the statistics software R (version 4.0.0, [48]), as well as the XLSTAT statistical and data analysis solution for Excel. Principal Component Analysis (PCA) was applied to detect chemical compounds that best discriminate between hemp samples in this study. A one-way analysis of variance (ANOVA) followed by the Tukey’ HSD test was carried out on total phenolic content and antioxidant activity for each hemp cultivar, and the level of significance was chosen as α = 0.05. Correlation analysis was performed using the Pearson criterion.

## Figures and Tables

**Table 1:** Tetrahydrocannabinolic acid (THCA), cannabidiolic acid (CBDA) and cannabigerolic acid (CBGA) concentrations on the last harvest date, for each hemp cultivar.

Hemp Cultivar	Sowing Density	Date of Harvest/Flowering Stage	THCA Concentration (mg/g hemp)	CBDA Concentration (mg/g hemp)	CBGA Concentration (mg/g hemp)
Finola ♀	300	11.08.2019 b	0.08 ± 0.001	1.99 ± 0.04	*ND*
Felina 32	300	10.09.2019 b	0.11 ± 0.012	3.41 ± 0.37	*ND*
Fibror 79	300	10.09.2019 a	0.13 ± 0.006	3.54 ± 0.10	0.067 ± 0.004
Futura 75	300	10.09.2019 b	0.12 ± 0.005	3.38 ± 0.16	0.055 ± 0.001
KC Virtus ♂	300	27.08.2019	0.09 ± 0.001	2.48 ± 0.02	0.057 ± 0.004
KC Virtus ♀	300	10.09.2019 a	0.10 ± 0.033	2.72 ± 0.09	0.039 ± 0.003
Santhica 27	300	10.09.2019 b	*ND*	0.04 ± 0.001 *	1.536 ± 0.044 *
Santhica 70	300	10.09.2019 b	*ND*	0.16 ± 0.001 *	1.240 ± 0.010 *
Fedora 17	150	10.09.2019 b	0.09 ± 0.008	2.54 ± 0.12	0.061 ± 0.009

ND—not detected; * *t*—Student test, *p* < 0.01 from all other cultivars; a—full flowering stage; b—end of flowering stage. ♀—female plants; ♂—male plants.

**Table 2:** Total phenolic content (TPC) and DPPH scavenging activity of hemp samples. TPC is expressed as milligram gallic acid equivalent per gram of dry sample (mg GAE/g *dw*), DPPH radical scavenging activity as percentage.

Cultivar *Harvest date*	Indication of Phenological Phase	Sowing Density	TPC [mg GEA/g sample]	% of DPPH Radical Scavenging Activity
**Fedora 17**				
22.07.2019	Early flowering	150	22.05 ± 0.46 a ^1^	43.35 ± 0.25 a
13.08.2019	Full flowering		10.5 ± 0.18 b	14.45 ± 5.71 b
27.08.2019	End of flowering		10.63 ± 0.27 b	22.76 ± 0.69 b
10.09.2019			9.36 ± 0.12 c	13.65 ± 5.53 b
**Felina 32**				
22.07.2019	Early flowering	300	21.97 ± 0.05 a	38.06 ± 0.13 a
10.09.2019	End of flowering		14.68 ± 0.17 c	21.06 ± 0.75 d
22.07.2019	Early flowering	150	17.14 ± 0.29 b	27.06 ± 0.38 b
13.08.2019	Full flowering		14.67 ± 0.21 c	22.09 ± 0.25 c d
27.08.2019	End of flowering		13.88 ± 0.25 d	24.40 ± 0.75 c
10.09.2019			8.40 ± 0.20 e	12.60 ± 0.31 e
**Fibror 79**				
22.07.2019	Early flowering	300	18.12 ± 0.21 a	32.77 ± 2.45 a
27.08.2019			13.37 ± 0.17 c	16.81 ± 1.13 d e
10.09.2019	Full flowering		7.26 ± 0.08 e	9.68 ± 0.25 f
22.07.2019	Early flowering	150	18.01 ± 0.15 a	29.77 ± 0.88 a b
13.08.2019			16.98 ± 0.13 b	27.99 ± 1.07 b
27.08.2019			11.23 ± 0.23 d	15.87 ± 0.44 e
10.09.2019	Full flowering		13.40 ± 0.10 c	19.53 ± 0.31 c d
13.08.2019	Early flowering	30	18.18 ± 0.34 a	29.36 ± 0.25 a b
27.08.2019			16.61 ± 0.18 a	27.20 ± 0.75 b
10.09.2019	Full flowering		13.02 ± 0.36 c	23.05 ± 0.06 c
**Finola**				
02.07.2019 ♂		150	10.77 ± 0.29 c	24.89 ± 0.13 b
02.07.2019 ♀	Full flowering		15.46 ± 0.22 a	31.10 ± 0.69 a
16.07.2019 ♀	End of flowering		14.28 ± 0.13 b	26.93 ± 0.19 b
30.07.2019 ♀			9.71 ± 0.58 c d	16.19 ± 0.57 c
11.08.2019 ♀			8.63 ± 0.72 d	13.65 ± 0.25 d
11.08.2019 ♀	End of flowering	300	5.38 ± 0.23 e	8.34 ± 0.82 e
**Futura 75**				
22.07.2019	Early flowering	300	16.11 ± 0.20 a	32.22 ± 0.38 a
13.08.2019			9.76 ± 0.14 d	11.72 ± 0.31 e
10.09.2019	End of flowering		8.26 ± 0.09 e	16.16 ± 0.38 d
22.07.2019	Early flowering	150	15.67 ± 0.17 a	27.04 ± 1.07 b
13.08.2019			14.90 ± 0.35 b	23.98 ± 0.13 c
27.08.2019	Full flowering		10.33 ± 0.12 c	17.85 ± 0.63 d
10.09.2019	End of flowering		7.85 ± 0.20 e	13.52 ± 0.38 e
**KC Virtus**				
13.08.2019 ♂		300	18.31 ± 0.94 b	33.36 ± 0.69 b c d
27.08.2019 ♂			11.50 ± 0.17 f	17.40 ± 0.25 f
22.07.2019 ♀	Early flowering		20.28 ± 0.59 a	33.50 ± 0.57 b c
13.08.2019 ♀			18.30 ± 0.15 b	33.26 ± 0.82 b
27.08.2019 ♀			16.55 ± 0.16 c	26.55 ± 0.75 d e
10.09.2019 ♀	Full flowering		4.80 ± 0.18 h	7.57 ± 0.63 g
13.08.2019 ♂		150	7.58 ± 0.21 g	9.66 ± 2.45 g
27.08.2019 ♂			16.05 ± 0.30 c d	25.09 ± 2.39 e
22.07.2019 ♀	Early flowering		15.16 ± 0.65 d e	27.33 ± 1.19 c d e
13.08.2019 ♀			16.39 ± 0.05 c d	24.61 ± 0.50 e
27.08.2019 ♀			16.49 ± 0.61 c d	30.31 ± 3.52 b c d e
10.09.2019 ♀	Full flowering		5.58 ± 0.08 h	8.34 ± 1.19 g
27.08.2019 ♂		30	14.54 ± 0.46 e	25.96 ± 1.44 e
27.08.2019 ♀	Early flowering		21.51 ± 0.57 a	40.15 ± 0.50 a
10.09.2019 ♀	Full flowering		15.76 ± 0.44 c d e	28.95 ± 0.94 b c d e
**Santhica 27**				
22.07.2019	Early flowering	300	17.14 ± 0.54 a	32.33 ± 1.76 a
13.08.2019	Full flowering		13.36 ± 0.21 b	23.21 ± 0.88 b
27.08.2019			6.45 ± 0.06 c	9.11 ± 1.07 c
10.09.2019	End of flowering		7.32 ± 0.06 d	11.52 ± 0.63 c
**Santhica 70**				
22.07.2019	Early flowering	300	16.46 ± 0.23 a	35.21 ± 1.00 a
13.08.2019	Full flowering		9.00 ± 0.42 b	10.00 ± 4.02 b
10.09.2019	End of flowering		4.72 ± 0.11 c	8.17 ± 0.44 b

^1^ Data were evaluated via one-way ANOVA separately for each cultivar, followed by a Tukey test. Groups with the same letter are not significantly different at α = 0.05; *dw* for dry weight; ♀ female plants; ♂ male plants.

**Table 3:** Mass spectrometric identification results.

No.	Attempted Identification	Abbr.	RT (min)	UV (nm)	Molecular Formula	MW (g/mol)	(M+H)^+^ (*m*/*z*)	Major Fragments (M+H)^+^(*m*/*z*)			Collision Energy (eV)
1 ♂	Quercetin-*O*-sophoroside	*Q-soph*	16.7	360	C_27_H_30_O_17_	626.517	627.157	303.050	465.104	-	15
2	Isoorientin-2’’-*O*-glucoside	*I-gluc*	18.4	360	C_27_H_30_O_16_	610.517	611.162	449.105	329.066	287.059	30
3	Luteolin-*C*-glucoside	*L-gluc*	18.7	360	C_21_H_20_O_11_	448.377	449.109	329.07	287.055	-	30
4	Luteolin-*C*-hexoside-*O*-rhamnoside	*L-hex-rhamn*	19.1	360	C_27_H_30_O_15_	594.518	595.167	449.108	329.068	287.054	30
5 ♂	Kaempferol-*O*-sophoroside	*K-soph*	20.0	360	C_27_H_30_O_16_	610.517	611.161	287.056	153.021; 121.029; 165.02 (70eV)	-	30
6	Vitexin-2’’-*O*-glucoside	*V-gluc*	21.3	360	C_27_H_30_O_15_	594.518	595.166	433.11	313.072	271.059	30
7	Vitexin-2’’-*O*-rhamnoside *	*V-rhamn*	22.6	360	C_27_H_30_O_14_	578.519	579.171	433.113	313.074	271.058	15
8	Luteolin-7-*O*-glucuronide *	*L-glucu*	25.7	360	C_21_H_18_O_12_	463.460	463.088	287.056	-	-	30
9	Apigenin-7-*O*-glucuronide *	*A-glucu*	28.7	320	C_21_H_18_O_11_	446.361	447.092	271.061	-	-	30
10	Chrysoeriol-*O*-glucuronide isomer 1	*C-glucu-1*	28.7	360	C_22_H_20_O_12_	476.387	477.104	301.071	286.047	258.050	45
11	Chrysoeriol-*O*-glucuronide isomer 2	*C-glucu-2*	28.8	360	C_22_H_20_O_12_	476.387	477.104	301.071	286.048	258.051	45
12	Apigenin-4’-methoxy-7-glucuronide	*A-meth-glucu*	29.4	320	C_22_H_20_O_11_	460.388	461.110	285.077	133.017	153.014	45
13	Luteolin *	*Lut*	29.4	360	C_15_H_10_O_6_	286.236	287.056	153.02	135.046	161.023	45
14	Apigenin *	*Api*	29.9	320	C_15_H_10_O_5_	270.237	271.066	153.019	145.028	121.03	45
15	Chrysoeriol *	*Chryso*	30.2	360	C_16_H_12_O_6_	300.260	301.072	286.048	258.054	-	30

* Identification confirmed by comparison with the reference standard; ♂ compounds isolated only in male plants.

**Table 4:** Observed clusters of hemp cultivars, and principal characteristics.

Group	Cultivar/Density/ Harvest Date/Flowering stage	Average Terpene Content (mg/kg)	Average Ratio Mono-/sesquiterpenes	Characteristic Terpenes
A	Santhica 70, 300, 13/08 (full flowering) Santhica 27, 300, 27/08 (full flowering) Finola, 150, 11/08 (end of flowering)	268.4 ± 40.4	0.9	Finola only: (*E*)-ocimene.Overall low amount of all terpene
B	Fedora 17, 150, 13/08 (full flowering) Felina 32, 150, 27/08 (end of flowering) Futura 75, 150, 27/08 (full flowering)	820.7 ± 171.9	2.3	Myrcene, Terpinolene, Beta-phellandrene, (*E*)-ocimene
C	KC Virtus, 150, 10/09 (full flowering) KC Virtus, 150, 27/08 (early flowering) Fibror 79, 30-150-300, 27/08 (early flowering)	631.9 ± 81.1	2.1	Limonene, Linalool, Alpha-bisabolol

**Table 5:** Main characteristics of the eight hemp cultivars cultivated in Switzerland in the present study.

Genotype	Origin	Characteristics	Species	Cultivated Density (Plants/m^2^)
Fedora 17	France	Average earliness	Monoecious	150
Felina 32	France	Average earliness	Monoecious	150/300
Futura 75	France	Average early maturing	Monoecious	150/300
Santhica 27	France	Average early maturing	Monoecious	300
Santhica 70	France	Average early maturing	Monoecious	300
Fibror 79	France	Late maturing	Monoecious	30/150/300
KC Virtus	Hungary	Late maturing	Dioecious	30/150/300
Finola	Finland	Early	Dioecious	150/300

**Table 6:** Monthly minimum temperature (T_min_), maximum temperature (T_max_), total rainfall and average humidity between May and September 2019 in Wädenswil-Obstbau (Switzerland) (Agrometeo, Agroscope, Wädenswil, Switzerland).

Month	T_min_ (°C)	T_max_ (°C)	Total Rainfall (mm)	Average Humidity (%)
May	0.5	23.3	142.5	75.5
June	9.9	36.4	89	67.7
July	11.9	36.2	181.6	67.3
August	10.9	32.3	159.2	77.6
September	6.6	28.5	131.8	81
Mean	8.0	31.3		73.8
Total			704.1	

**Table 7:** Phenological stage of each cultivar at each harvest date.

Cultivar/Harvest Date	22.07.2019	13.08.2019	27.08.2019	10.09.2019
Futura 75	early flowering	early flowering	full flowering	end of flowering
Felina 32	early flowering	full flowering	end of flowering	end of flowering
Fedora 17	early flowering	full flowering	end of flowering	end of flowering
Santhica 27	early flowering	full flowering	full flowering	end of flowering
Santhica 70	early flowering	full flowering	full flowering	end of flowering
Fibror 79	early flowering	early flowering	early flowering	full flowering
KC Virtus	early flowering	early flowering	early flowering	full flowering
**Cultivar/Harvest Date**	**02.07.2019**	**16.07.2019**	**30.07.2019**	**11.08.2019**
Finola	full flowering	end of flowering	end of flowering	end of flowering

**Table 8:** Linear equations of the calibration lines of each reference substance used in the study.

Compound	UV (nm)	Range (mg/mL)	Linear Equation	R^2^
Vitexin-2-*O*-rhamnoside	360	0.1–1	y = 9799.6x + 409.54	0.9984
Luteolin-7-*O*-glucuronide	360	0.1–1	y = 14862x + 352.91	0.9989
Apigenin-7-*O*-glucuronide	320	0.1–0.5	y = 18766x + 852.21	0.9892
Apigenin	320	0.01–0.25	y = 27617x + 69.461	0.9982
Kaempferol	360	0.01–0.25	y = 36241x − 51.284	0.9995
Luteolin	360	0.01–0.25	y = 29449x + 71.492	0.9974
Quercetin	360	0.01–0.25	y = 25592x + 118.05	0.996
Chrysoeriol	360	0.01–0.25	y = 30287x + 43.037	0.9981
Tetrahydrocannabinolic acid (THCA)	280	0.05–0.1	y = 10402x − 4.3312	0.9998
Cannabiodiolic acid (CBDA)	280	0.025–1	y = 8751.7x + 72.234	0.9936
Cannabigerolic acid (CBGA)	280	0.025–1	y = 8050.9x + 34.236	0.9994

## Supplementary Materials

The following are available online at https://www.mdpi.com/2223-7747/9/12/1740/s1, Table S1: Morphological parameters of the different cultivars sowed at densities: 30, 150 and 300 seeds/m2; Table S2: Correlation matrix with Pearson coefficient values for the total phenolic content (TPC), the different identified flavonoids and the antioxidant activity (AOX) of the hemp extracts; Table S3: Maximum absorbance wavelength of reference compounds, and compounds identified in the different hemp cultivars; Table S4: Flavonoid composition of Fedora 17 density 150. Data are presented as mean ± SD in mg/g DW; Table S5; Flavonoid composition of Felina 32 density 300 and 150 seeds/m^2^. Data are presented as mean ± SD in mg/g DW; Table S6: Flavonoid composition of KC Virtus density 30 seeds/m^2^, male and female inflorescences. Data are presented as mean ± SD in mg/g DW; Table S7: Flavonoid composition of KC Virtus density 150 seeds/m^2^, male and female inflorescences. Data are presented as mean ± SD in mg/g DW; Table S8: Flavonoid composition of KC Virtus density 300 seeds/m^2^, male and female inflorescences. Data are presented as mean ± SD in mg/g DW; Table S9: Flavonoid composition of Fibror 79 density 300, 150 and 30 seeds/m^2^ inflorescences. Data are presented as mean ± SD in mg/g DW; Table S10: Flavonoid composition of Santhica 27 density 300 seeds/m^2^ inflorescences. Data are presented as mean ± SD in mg/g DW; Table S11: Flavonoid composition of Futura 75 density 300 and 150 seeds/m^2^ inflorescences. Data are presented as mean ± SD in mg/g DW; Table S12: Flavonoid composition of Santhica 70 density 300 seeds/m^2^ inflorescences. Data are presented as mean ± SD in mg/g DW; Table S13: Flavonoid composition of Finola density 300 and 150 seeds/m^2^, male and female inflorescences. Data are presented as mean ± SD in mg/g DW; Table S14: Qualitative analysis of the different terpenes found in 8 different hemp varieties; Table S15: Terpene composition of different hemp cultivars sowed at different densities and harvested at different dates. Data are expressed as mean ± SD in mg/kg FW, flowering stages are indicated by the letters: a = early flowering, b = full flowering, c = end of flowering.; Figure S1: MS/MS spectra of Quercetin-*O*-sophoroside (1), CEV = 30 eV, (M+H)+ = 627.15655; Figure S2: MS/MS spectra of Isoorientin-2’’-*O*-glucoside (2), CEV = 30 eV, (M+H)+ = 611.16136; Figure S3: MS/MS spectra of Luteolin-C-glucoside (3), CEV = 30 eV, (M+H)+ = 449.10887; Figure S4: MS/MS spectra of Luteolin-*C*-hexoside-*O*-rhamnoside (4), CEV = 30 eV, (M+H)+ = 595.16658; Figure S5: MS/MS spectra of Kaempferol-*O*-sophoroside (5), CEV = 30 eV, (M+H)+ = 611.16139; Figure S6: MS/MS spectra of Vitexin-2’’-*O*-glucoside (6), CEV = 30 eV, (M+H)+ = 595.166510; Figure S7: MS/MS spectra of Vitexin-2’’-*O*-rhamnoside (7), CEV = 30 eV, (M+H)+ = 579.171711; Figure S8: MS/MS spectra of Luteolin-7-*O*-glucuronide (8), CEV = 30 eV, (M+H)+ = 463.088412; Figure S9: MS/MS spectra of Apigenin-4’-methoxy-7-glucuronide (12), CEV = 45 eV, (M+H)+ = 461.108413; Figure S10: UV spectra of compound 3 (luteolin-C-glucoside), in comparison with UV spectra of Luteolin (blue) and Kaempferol (brown); Figure S11: UV spectra of compound 4 (luteolin-*C*-hexoside-*O*-rhamnoside), in comparison with UV spectra of Luteolin (blue) and Kaempferol (brown).
