# Supplementary material for: Evolution of the Polyphenol and Terpene Content, Antioxidant Activity and Plant Morphology of Eight Different Fiber-Type Cultivars of Cannabis sativa L. Cultivated at Three Sowing Densities
Source: Plants (Basel). 2020 Dec 9;9(12):1740. doi: 10.3390/plants9121740 (PMC7763896; doi:10.3390/plants9121740)
Supplement: Supplementary file 1 [file plants-09-01740-s001.pdf]

plants

MDPI

SUPPLEMENTARY MATERIAL

Evolution of the polyphenol and terpene content, antioxidant activity and plant morphology during the ripening of eight different fiber-type cultivars of *Cannabis sativa* L., and influence of the sowing density

Amandine André *, Marianne Leupin, Markus Kneubühl, Vasilisa Pedan and Irene Chetschik

Zurich University of Applied Sciences, Life Sciences and Facility Management, 8820 Wädenswil, Switzerland

Table S 1. Morphological parameters of the different cultivars sowed at densities: 30, 150 and 300 seeds/m² 3
Table S 2. Correlation matrix with Pearson coefficient values for the total phenolic content (TPC), the different identified flavonoids and the antioxidant activity (AOX) of the hemp extracts. 6
Table S 3. Maximum absorbance wavelength of reference compounds, and compounds identified in the different hemp cultivars. 16
Table S 4. Flavonoid composition of Fedora 17 density 150. Data are presented as mean ± SD in mg/g DW. 19
Table S 5. Flavonoid composition of Felina 32 density 300 and 150 seeds/m². Data are presented as mean ± SD in mg/g DW 20
Table S 6. Flavonoid composition of KC Virtus density 30 seeds/m², male and female inflorescences. Data are presented as mean ± SD in mg/g DW 21
Table S 7. Flavonoid composition of KC Virtus density 150 seeds/m², male and female inflorescences. Data are presented as mean ± SD in mg/g DW 22
Table S 8. Flavonoid composition of KC Virtus density 300 seeds/m², male and female inflorescences. Data are presented as mean ± SD in mg/g DW 23
Table S 9. Flavonoid composition of Fibror 79 density 300, 150 and 30 seeds/m² inflorescences. Data are presented as mean ± SD in mg/g DW 24
Table S 10. Flavonoid composition of Santhica 27 density 300 seeds/m² inflorescences. Data are presented as mean ± SD in mg/g DW 26
Table S 11. Flavonoid composition of Futura 75 density 300 and 150 seeds/m² inflorescences. Data are presented as mean ± SD in mg/g DW 27
Table S 12. Flavonoid composition of Santhica 70 density 300 seeds/m² inflorescences. Data are presented as mean ± SD in mg/g DW 28
Table S 13. Flavonoid composition of Finola density 300 and 150 seeds/m², male and female inflorescences. Data are presented as mean ± SD in mg/g DW 29
Table S 14. Qualitative analysis of the different terpenes found in 8 different hemp varieties 30

Table S 15. Terpene composition of different hemp cultivars sowed at different densities and harvested at different dates. Data are expressed as mean ± SD in mg/kg FW, flowering stages are indicated by the letters: a = early flowering, b = full flowering, c = end of flowering........ 31

Figure S 1. MS/MS spectra of Quercetin-O-sophoroside (1), CEV = 30 eV, [M+H]$^+$ = 627.1565. 7
Figure S 2. MS/MS spectra of Isoorientin-2′′-O-glucoside (2), CEV = 30 eV, [M+H]$^+$ = 611.1613 8
Figure S 3. MS/MS spectra of Luteolin-C-glucoside (3), CEV = 30 eV, [M+H]$^+$ = 449.1088......... 9
Figure S 4. MS/MS spectra of Luteolin-C-hexoside-O-rhamnoside (4), CEV = 30 eV, [M+H]$^+$ = 595.1665 10
Figure S 5. MS/MS spectra of Kaempferol-O-sophoroside (5), CEV = 30 eV, [M+H]$^+$ = 611.1613 11
Figure S 6. MS/MS spectra of Vitexin-2′′-O-glucoside (6), CEV = 30 eV, [M+H]$^+$ = 595.1665... 12
Figure S 7. MS/MS spectra of Vitexin-2′′-O-rhamnoside (7), CEV = 30 eV, [M+H]$^+$ = 579.171713
Figure S 8. MS/MS spectra of Luteolin-7-O-glucuronide (8), CEV = 30 eV, [M+H]$^+$ = 463.0884 14
Figure S 9. MS/MS spectra of Apigenin-4′-methoxy-7-glucuronide (12), CEV = 45 eV, [M+H]$^+$ = 461.1084 15
Figure S 10. UV spectra of compound **3** (luteolin-C-glucoside), in comparison with UV spectra of Luteolin (blue) and Kaempferol (brown)... 17
Figure S 11. UV spectra of compound **4** (luteolin-C-hexoside-O-rhamnoside), in comparison with UV spectra of Luteolin (blue) and Kaempferol (brown).. 18

Table 1. Morphological parameters of the different cultivars sowed at densities: 30, 150 and 300 seeds/m^2.

Cultivar, Harvest Date and Flowering Stage	30 Seeds/m^2					150 Seeds/m^2					300 Seeds/m^2				
	% leaves	% flowers	% stem	Stem length (cm)	Stem diameter (cm)	% leaves	% flowers	% stem	Stem length (cm)	Stem diameter (cm)	% leaves	% flowers	% stem	Stem length (cm)	Stem diameter (cm)
**KC Virtus ♀**															
22.07.2019 *early flowering*						16.9	14.8	65	163.24 (a)	10.05 (a)	17.1	12.4	66.49	108.71 (b)	5.84 (b)
13.08.2019						15.1	18	66.91	144.54 (c)	nd	11.2	14.4	68.1	114.53 (d)	nd
27.08.2019	16.7	26.4	53.33	105.5 (e)		13	14.1	70	124.58 (f)	nd	12.8	15.3	70.7	145.75 (g)	nd
10.09.2019 *full flowering*	11.1	47.2	39.69	173.9 (h)	11.13	9.7	35.8	52.41	188.24 (h)	10.45	7.1	31.6	59.19	189.5 (h)	9.18
**Finola**															
02.07.2019 *full flowering*							61.2	28.7	65.5 (i)	4.05 (i)		61.4	34.6	60.4 (j)	3.84 (j)
16.07.2019 *end of flowering*							68.6	25.4	63.8 (k)	4.45 (k)		67.7	27.3	56.7 (l)	3.53 (l)
30.07.2019		65.88	21.42	72.8	5.39		68.8	32.98	99.94 (h)	5.91 (h)		65.33	27.2	66.48 (h)	4.25 (h)
11.08.2019	1.66	61.59	27.35	98.5		5.1	60.82	25.2	86.41		2.88	64.84	28.77	89	
25.08.2019	0.87	60.52	35.83	87.11		3.07	55.98	38.14	92.67		2.24	52.34	37.9	95.04	
**Felina 32**															
22.07.2019 *early flowering*						13.9	19.7	65.68	168.1	8.21	13.3	20.1	62.29	135.95	5.76
13.08.2019 *full flowering*						16.1	30	53.9	158.16		10.9	27.5	58.7	121.47	

27.08.2019 *end of flowering*	13.7	38.6	45.4	127.05		14	33.3	50.4	176.75		10.7	31.8	54.6	158.65	
10.09.2019	7	54.4	37.6	179.6	9.9	6.4	30.5	60.5	181.28	7.06	4.8	31.2	61	175.44	7.23
**Futura75**															
22.07.2019 *early flowering*						15.2	19.2	60.38	181.05	9.73	11.4	13.6	74.04	141.52	5.91
13.08.2019 *early flowering*						16.4	21.2	62.38	175.44		10.1	19.3	66.67	123.51	
27.08.2019 *full flowering*	13.8	32.4	50.98	122.79		12.2	26.8	59.38	167.14		9.9	23.9	64.35	166.95	
10.09.2019 *end of flowering*	2.7	39	56.25	180.4	10.3	3.8	25.2	68.46	181.12	7.14	1.5	25	70.55	192.88	9.07
**Fibror 79**															
22.07.2019 *early flowering*						14.5	13.8	68.49	154.75	7.54	12.4	13.1	72.96	146.67	6.28
13.08.2019						17	18.7	59.48	160		11.2	17.7	68.99	124	
27.08.2019	13.8	28.5	54.77	140		14.8	21.6	62.1	174		15	19	63.8	150	
10.09.2019 *full flowering*	10.8	36.7	49.23	191	12.9	9.5	27.8	60.2	199	9.2	9.1	33.6	55.33	196.95	10.15
**Fedora 17**															
22.07.2019 *early flowering*						16.6	25.5	53.4	141	7.67					
13.08.2019 *full flowering*						9.3	36.9	49.9	153.9						
27.08.2019 *end of flowering*						8.1	37.3	51.8	168						
10.09.2019						7.3	43.7	46.85	176.4	9.67					
**Santhica 27**															

22.07.2019 *early flowering*	13.2	16.5	63.87	161.8	7.66
13.08.2019 *full flowering*	13.3	22.8	63.8	146.3	
27.08.2019	9.9	26	61.7	172.2	
10.09.2019 *end of flowering*	3.2	23.6	70.6	187.6	7.4
**Santhica 70**					
22.07.2019 *early flowering*	14.4	15.9	66.9	167.3	9.13
13.08.2019 *full flowering*	14.2	19.5	66.37	159	
27.08.2019	11.3	23.2	63.64	155.38	
10.09.2019 *end of flowering*	4.6	18.1	75.2	176.92	7.04

Table 2. Correlation matrix with Pearson coefficient values for the total phenolic content (TPC), the different identified flavonoids and the antioxidant activity (AOX) of the hemp extracts.

Parameters	Q-soph	I-gluc	L-gluc	L-hex-rhamn	K-soph	V-gluc	V-rhamn	L-glucu	A-glucu	C-glucu-1	C-glucu-2	A-meth-glucu	Lut	Api	Chryso	TPC	AOX
Quercetine-O-sophoroside	1	0.19	0.12	0.10	0.97*	0.20	0.14	0.08	-0.01	0.18	0.33*	-0.12	0.12	0.41*	0.07	0.13	0.13
Isoorientin-2''-*O*-glucoside		1	0.93*	0.91*	0.18	0.90*	0.80*	0.85*	0.59*	0.70*	0.26	0.24	-0.16	-0.03	-0.13	0.73*	0.76*
Luteolin-*C*-glucoside			1	0.85*	0.12	0.90*	0.85*	0.85*	0.68*	0.74*	0.33*	0.31*	-0.10	-0.01	-0.08	0.70*	0.72*
Luteolin-*C*-hexoside-*O*-rhamnoside				1	0.08	0.79*	0.86*	0.91*	0.66*	0.73*	0.16	0.33*	-0.16	-0.09	-0.12	0.77*	0.81*
Kaempferol-*O*-sophoroside					1	0.19	0.13	0.07	-0.02	0.17	0.33*	-0.12	0.23	0.55*	0.15	0.12	0.12
Vitexin-2''-*O*-glucoside						1	0.91*	0.84*	0.77*	0.76*	0.46*	0.14	-0.12	0.00	-0.10	0.67*	0.67*
Vitexin-2′′-*O*-rhamnoside							1	0.89	0.87*	0.81*	0.38*	0.25	-0.07	0.00	-0.06	0.69*	0.70*
Luteolin-7-*O*-glucuronide								1	0.87*	0.86*	0.34*	0.30*	-0.18	-0.10	-0.13	0.80*	0.81*
Apigenin-7-*O*-glucuronide									1	0.83*	0.45*	0.19	-0.17	-0.12	-0.13	0.65*	0.63*
Chrysoeriol-*O*-glucuronide isomer 1										1	0.25	0.17	-0.15	-0.06	-0.12	0.75*	0.71*
Chrysoeriol-*O*-glucuronide isomer 2											1	-0.24	0.08	0.16	0.03	0.25	0.26
Apigenin-4'-methoxy-7-glucuronide												1	-0.09	-0.10	-0.06	0.13	0.09
Luteolin													1	0.89*	0.87*	-0.18	-0.21
Apigenin														1.00	0.80*	-0.10	-0.12
Chrysoeriol															1.00	-0.19	-0.20
TPC																1.00	0.96*
AOX																	1.00

*Correlation is significant at $p < 0.05$.

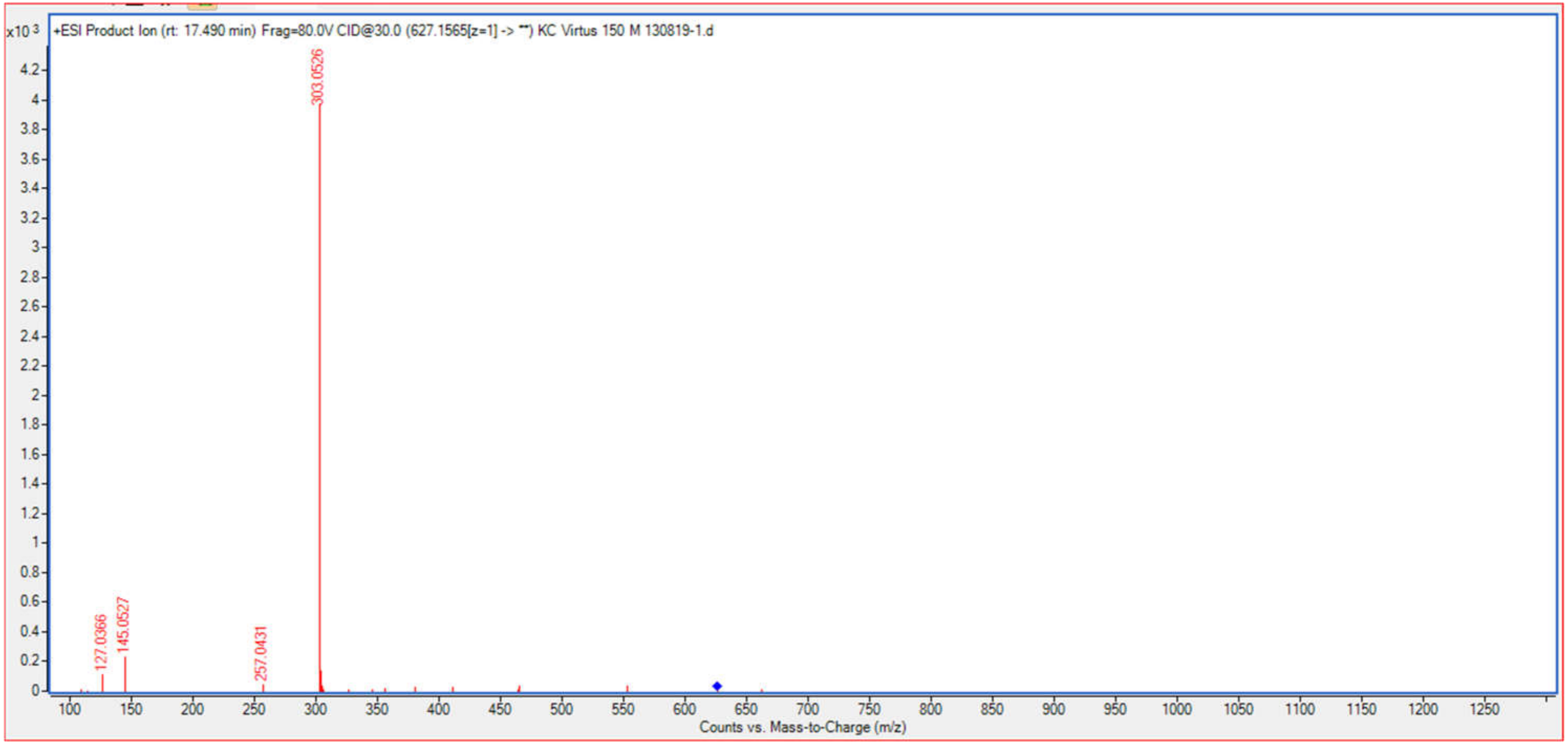

Figure 1. MS/MS spectra of Quercetin-O-sophoroside (1), CEV = 30 eV, $[M+H]^+$ = 627.1565.

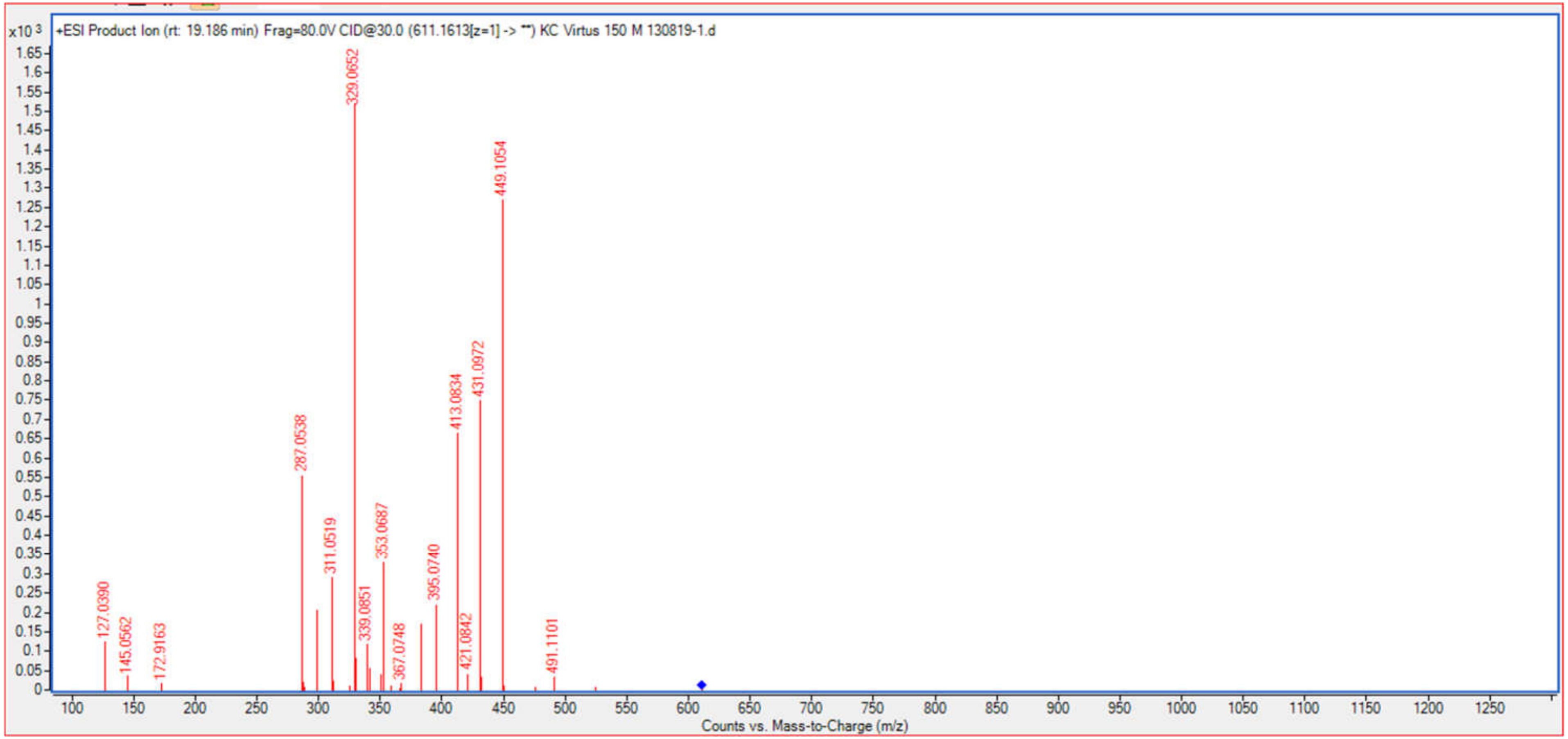

Figure 2. MS/MS spectra of Isoorientin-2′′-O-glucoside (2), CEV = 30 eV, $[M+H]^+$ = 611.1613.

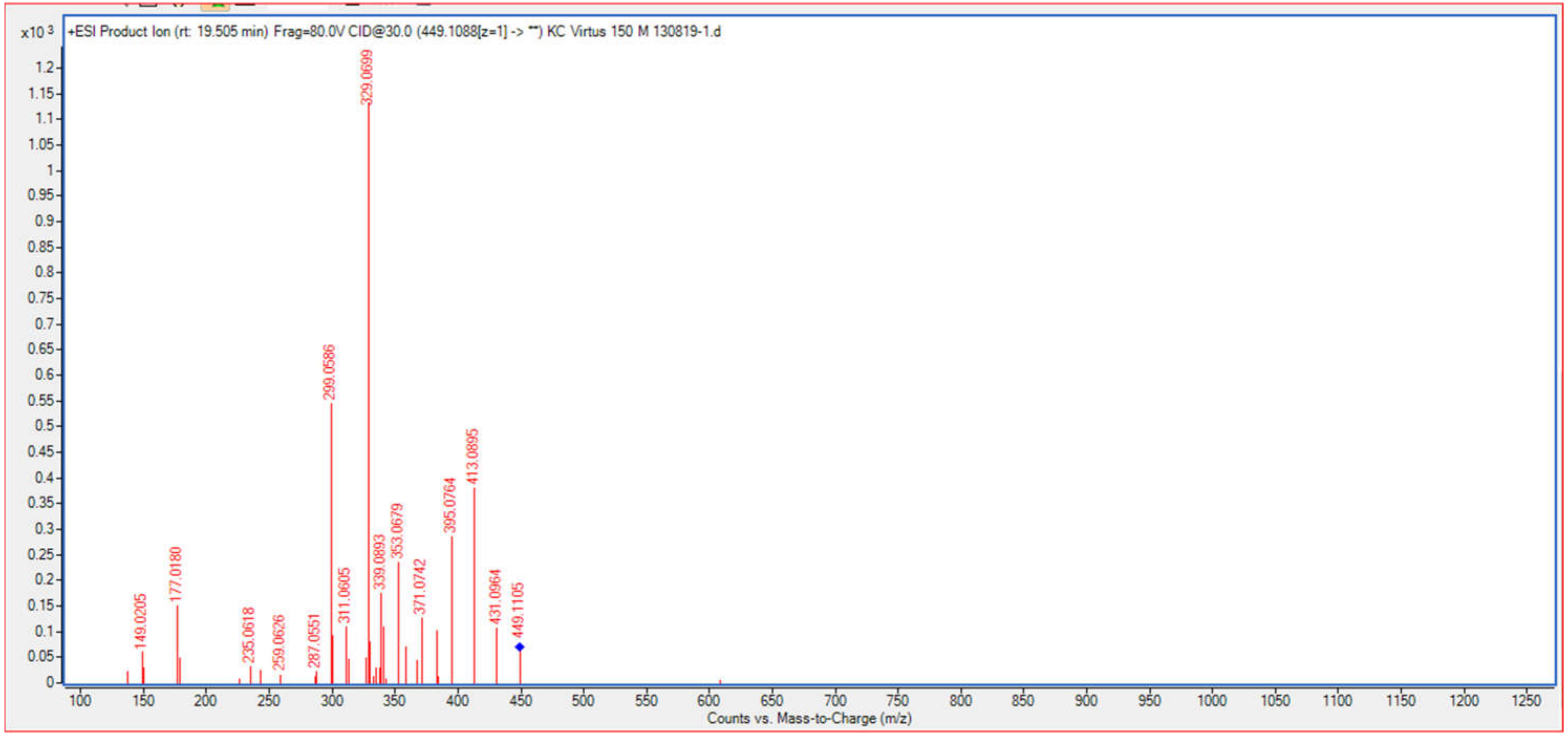

Figure 3. MS/MS spectra of Luteolin-C-glucoside (3), CEV = 30 eV, $[M+H]^+$ = 449.1088.

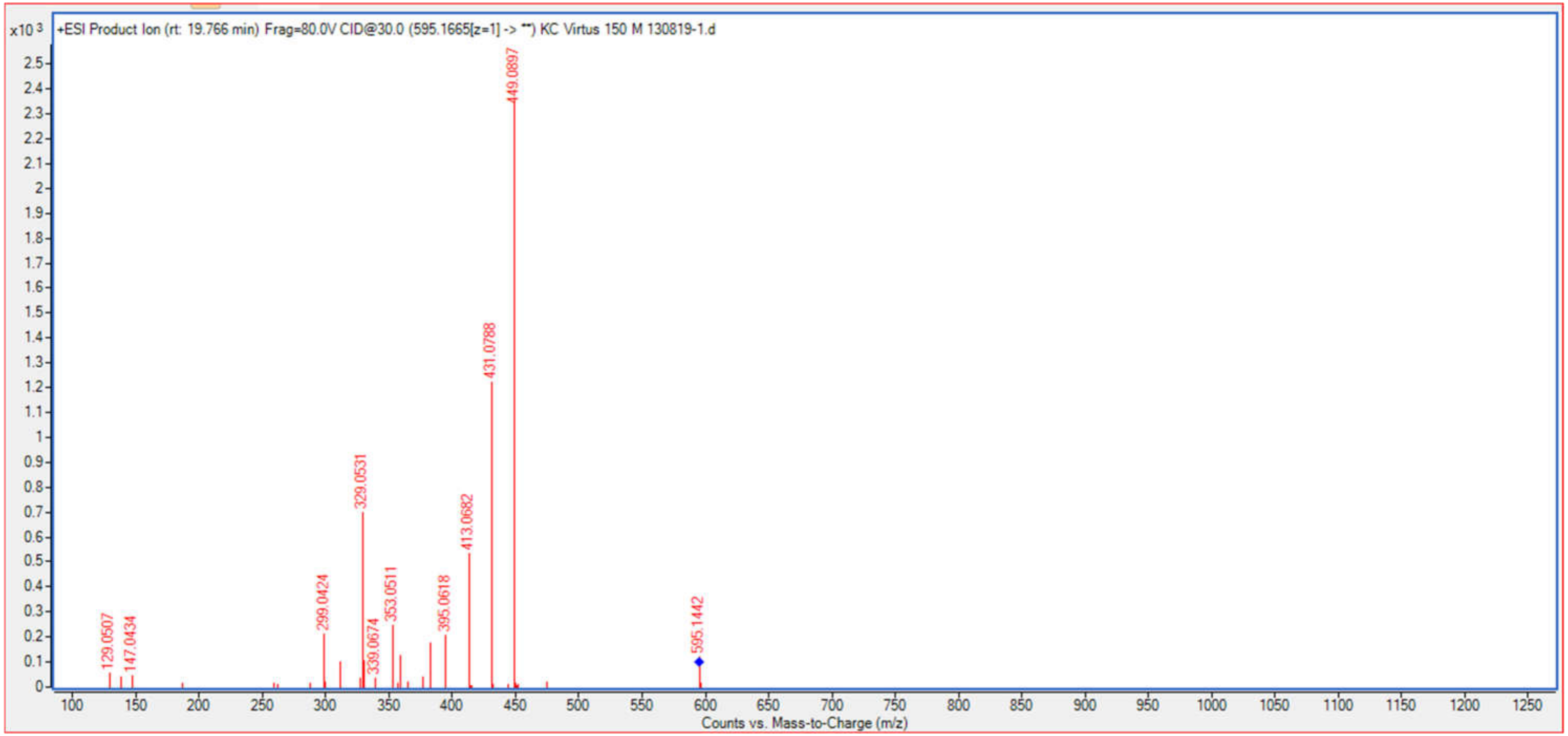

Figure 4. MS/MS spectra of Luteolin-C-hexoside-O-rhamnoside (4), CEV = 30 eV, $[M+H]^+$ = 595.1665.

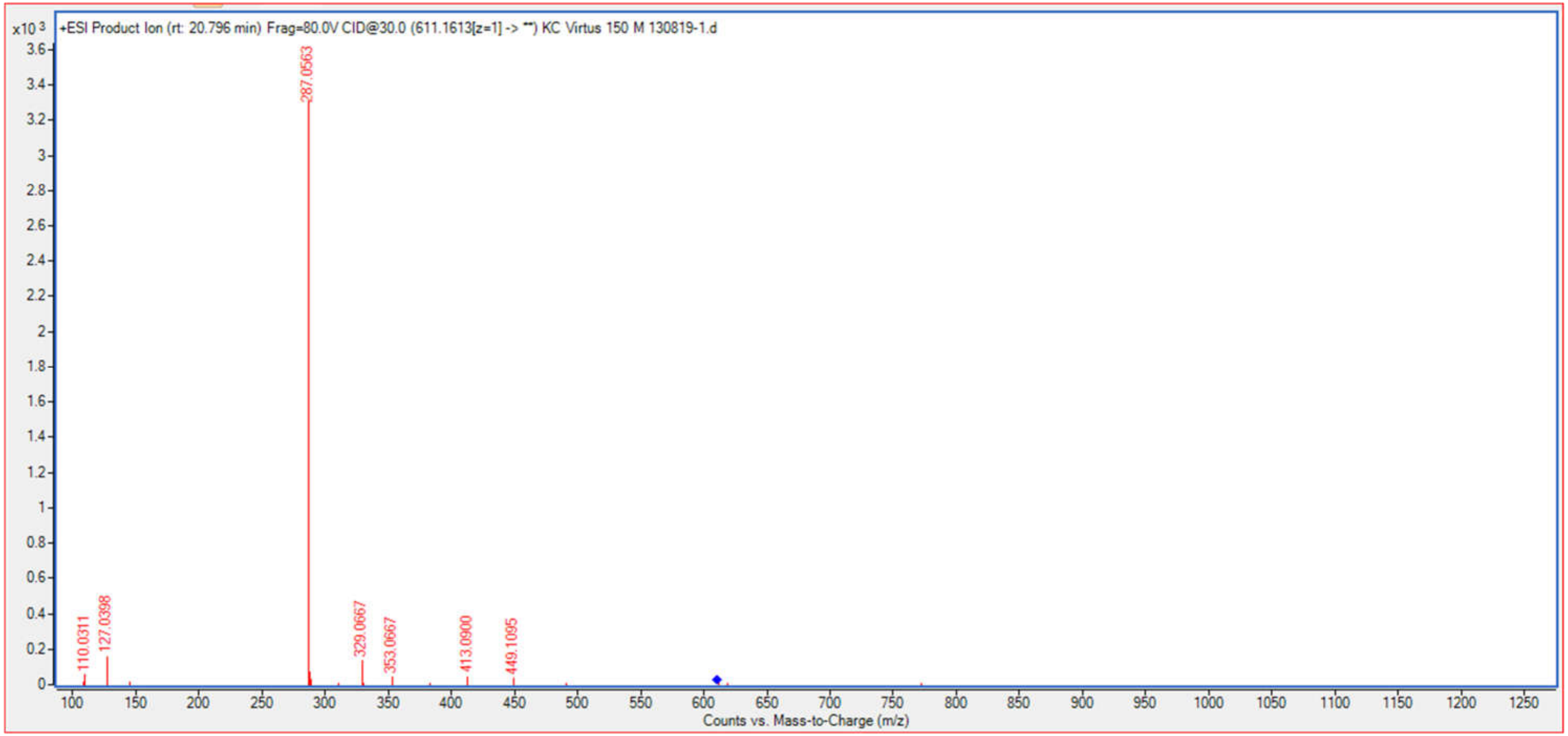

Figure 5. MS/MS spectra of Kaempferol-O-sophoroside (5), CEV = 30 eV, $[M+H]^+$ = 611.1613.

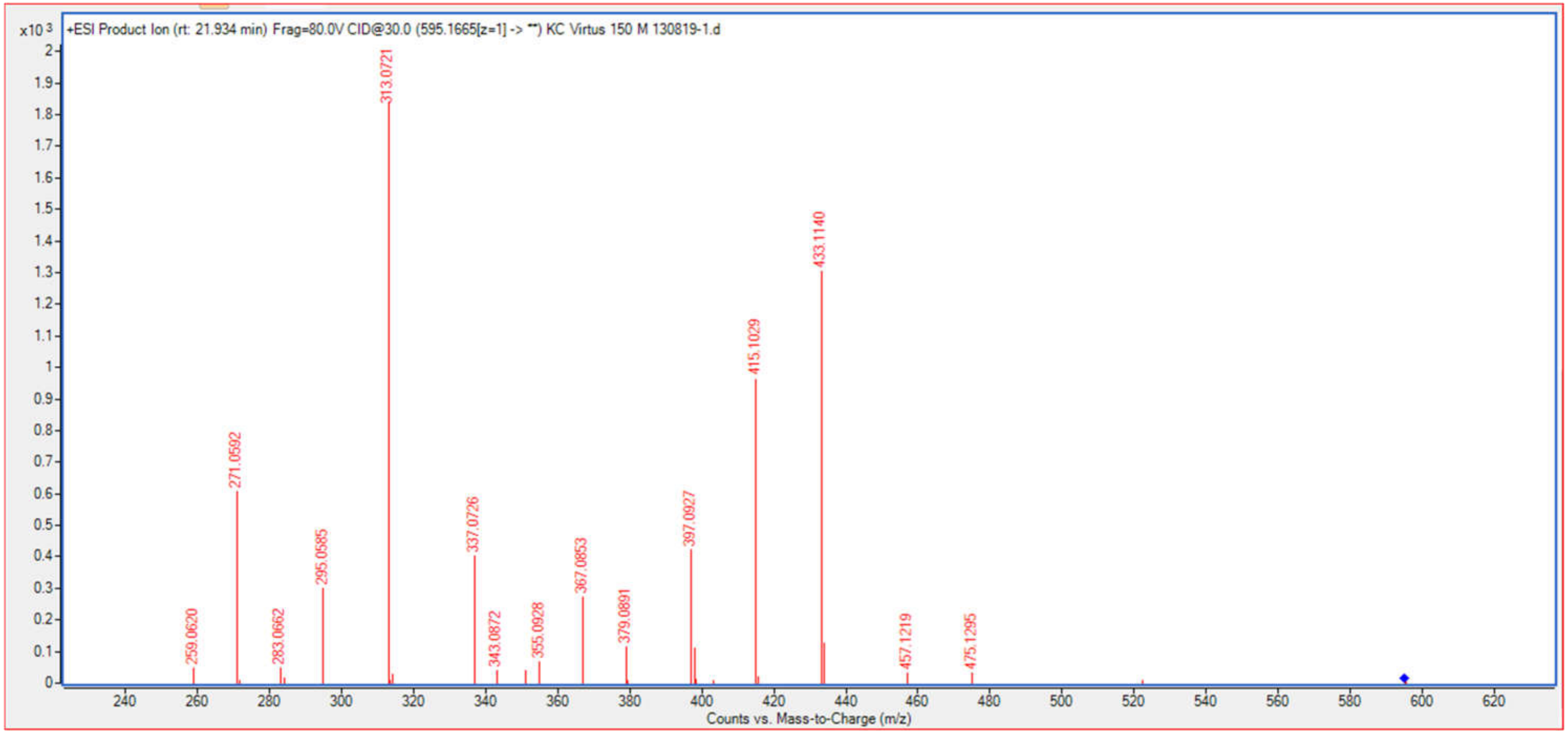

Figure 6. MS/MS spectra of Vitexin-2′′-O-glucoside (6), CEV = 30 eV, $[M+H]^+$ = 595.1665.

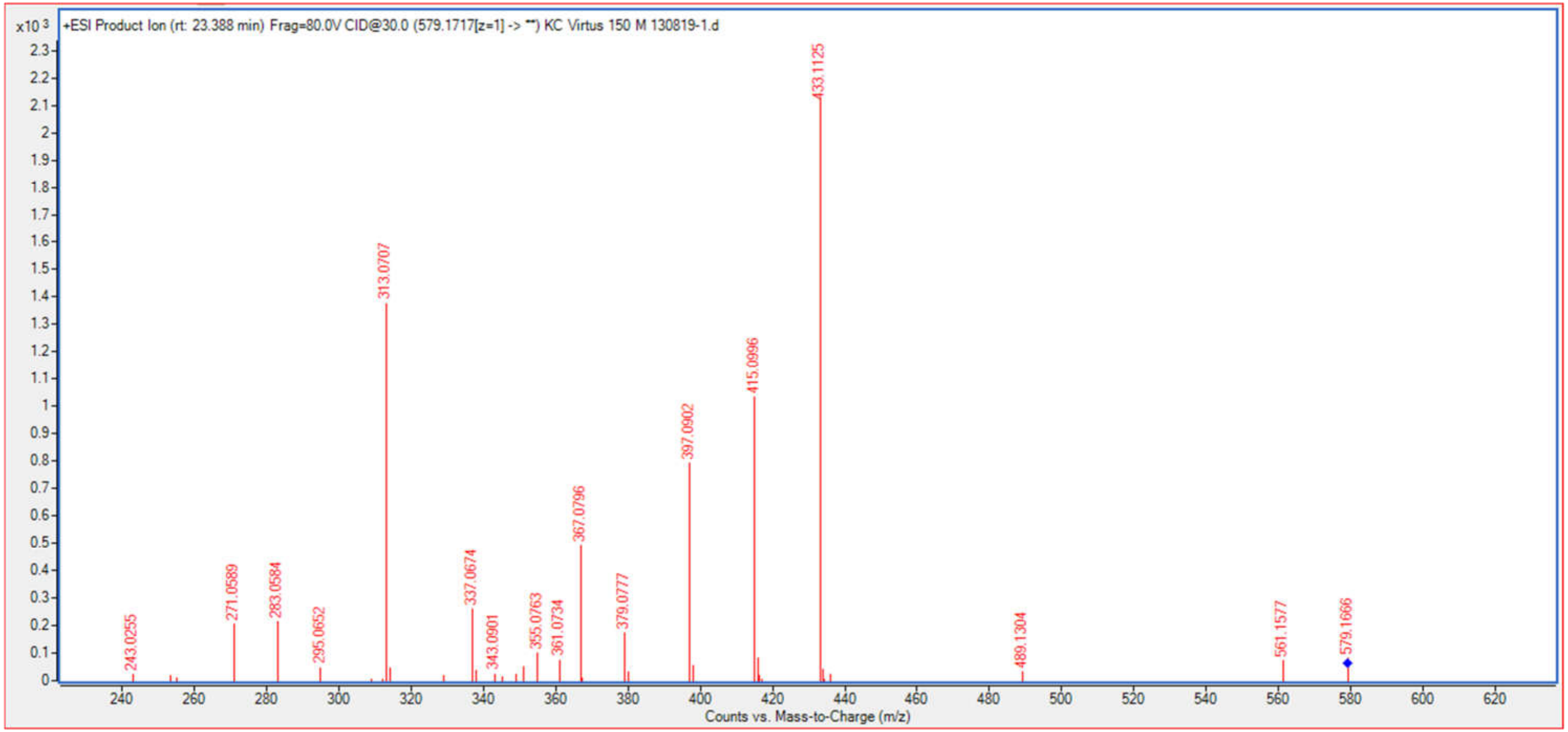

Figure 7. MS/MS spectra of Vitexin-2′′-O-rhamnoside (7), CEV = 30 eV, $[M+H]^+$ = 579.1717.

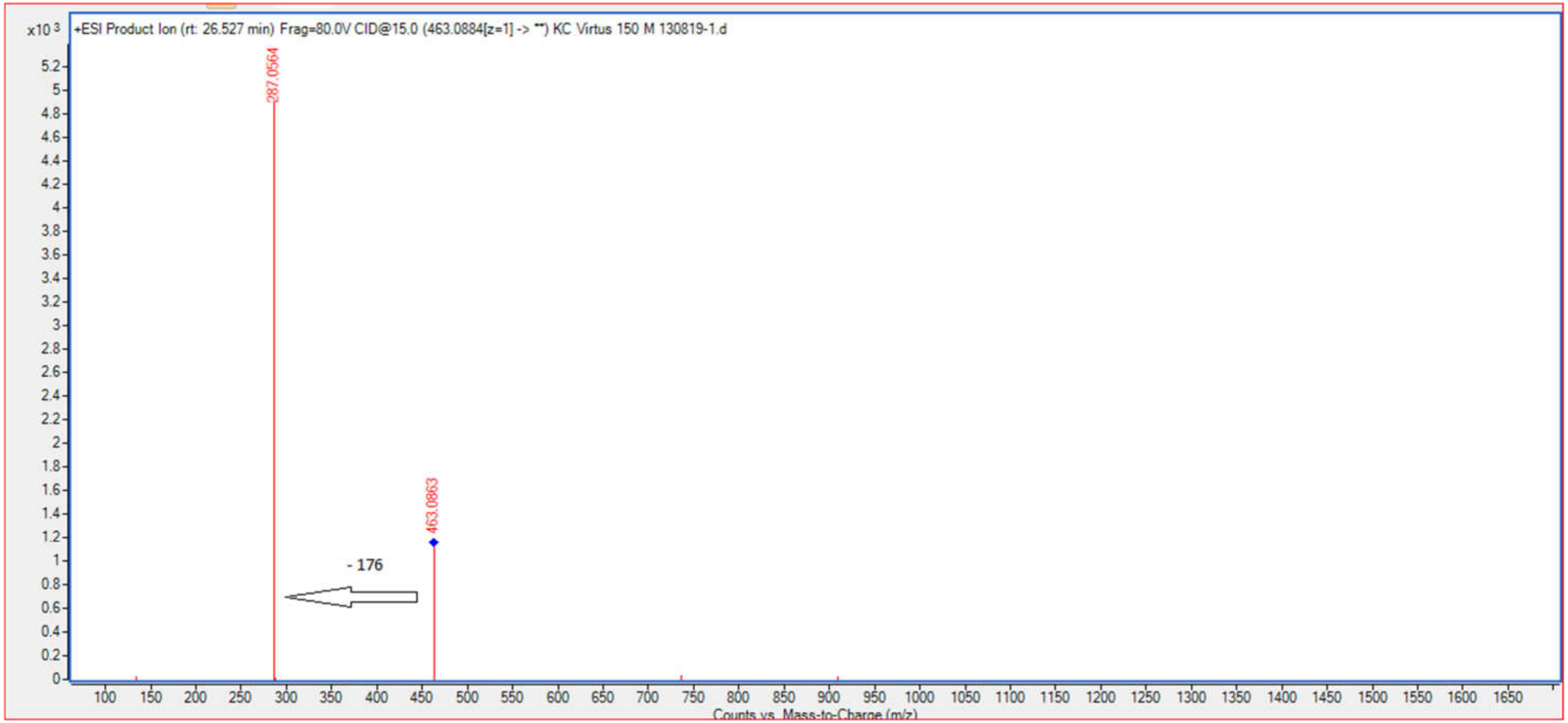

Figure 8. MS/MS spectra of Luteolin-7-O-glucuronide (8), CEV = 30 eV, $[M+H]^+$ = 463.0884.

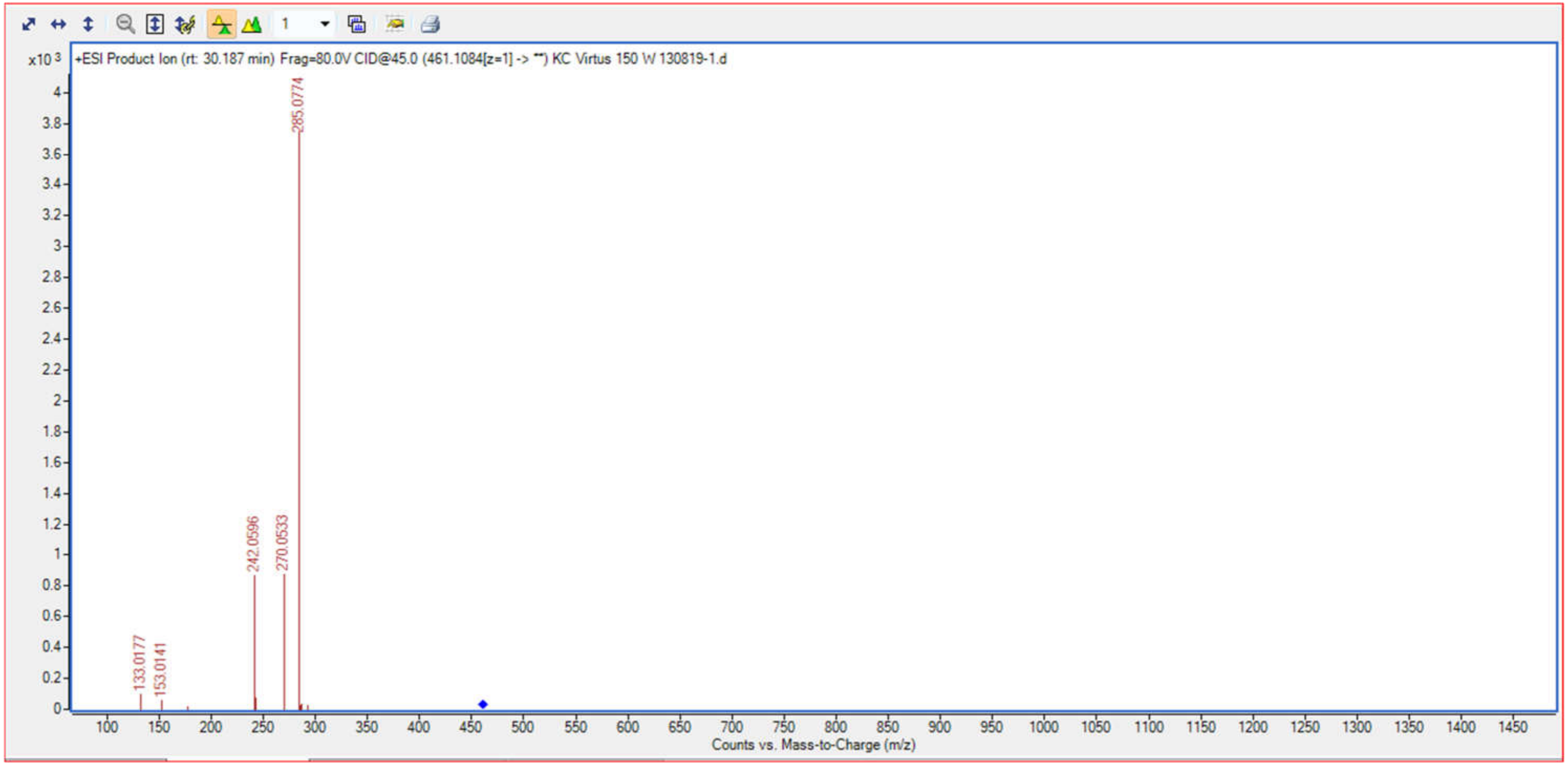

Figure 9. MS/MS spectra of Apigenin-4′-methoxy-7-glucuronide (12), CEV = 45 eV, $[M+H]^+$ = 461.1084.

Table 3. Maximum absorbance wavelength of reference compounds, and compounds identified in the different hemp cultivars.

**Compounds No,**	**Identification**	**λmax (nm)**		
Ref	Kaempferol	265	365	
Ref	Luteolin	252	267	349
Ref	Apigenin	268	338	
2	Isoorientin-2''-*O*-glucoside	254	266	349
3	Luteolin-*C*-glucoside	255	267	350
4	Luteolin-*C*-hexoside-*O*-rhamnoside	256	267	350
5	Kaempferol-sophoroside	265	346	
6	Vitexin-2''-*O*-glucoside	266	338	
7	Vitexin-2-*O*-rhamnoside *	266	338	

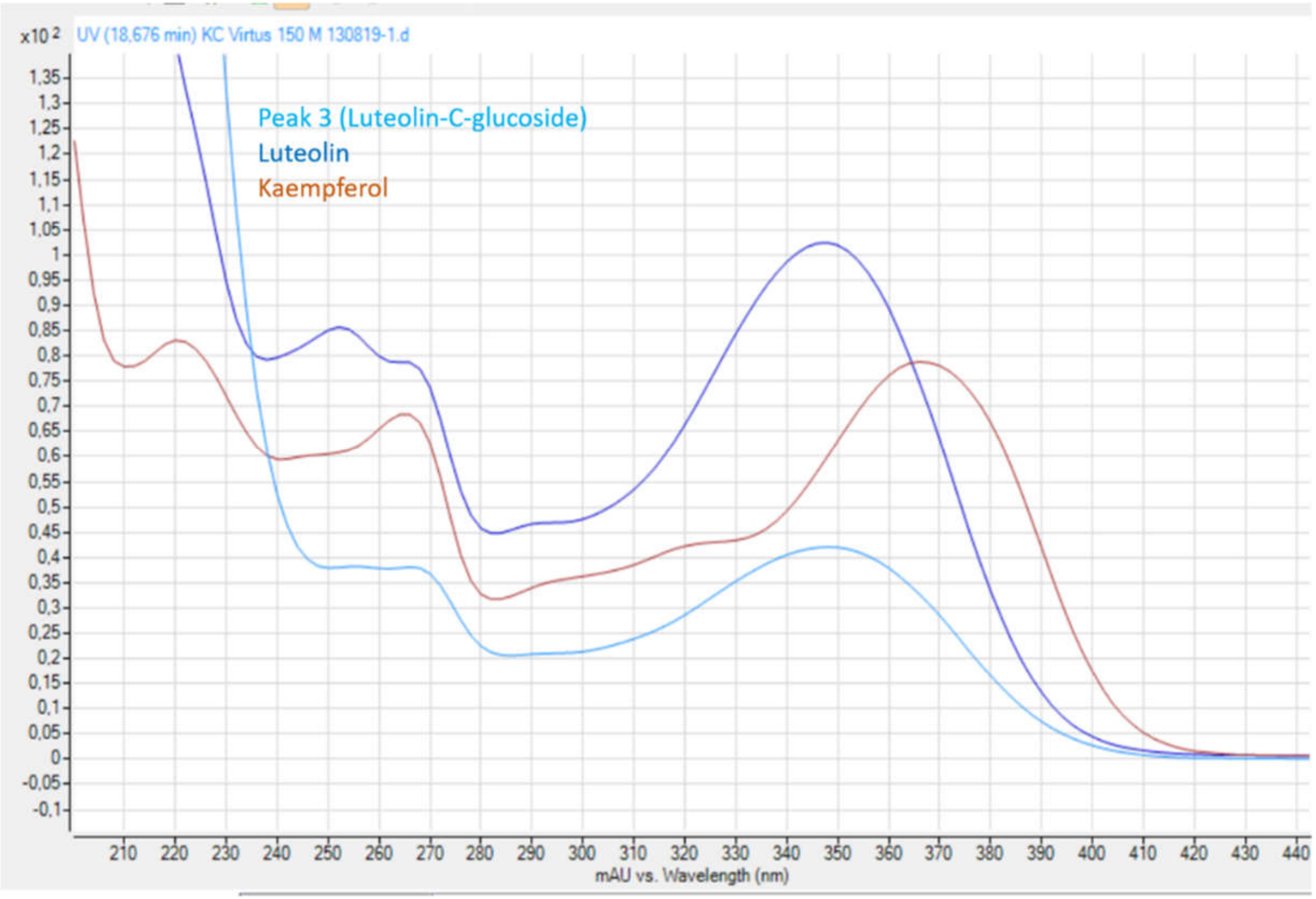

Figure 10. UV spectra of compound **3** (luteolin-C-glucoside), in comparison with UV spectra of Luteolin (blue) and Kaempferol (brown).

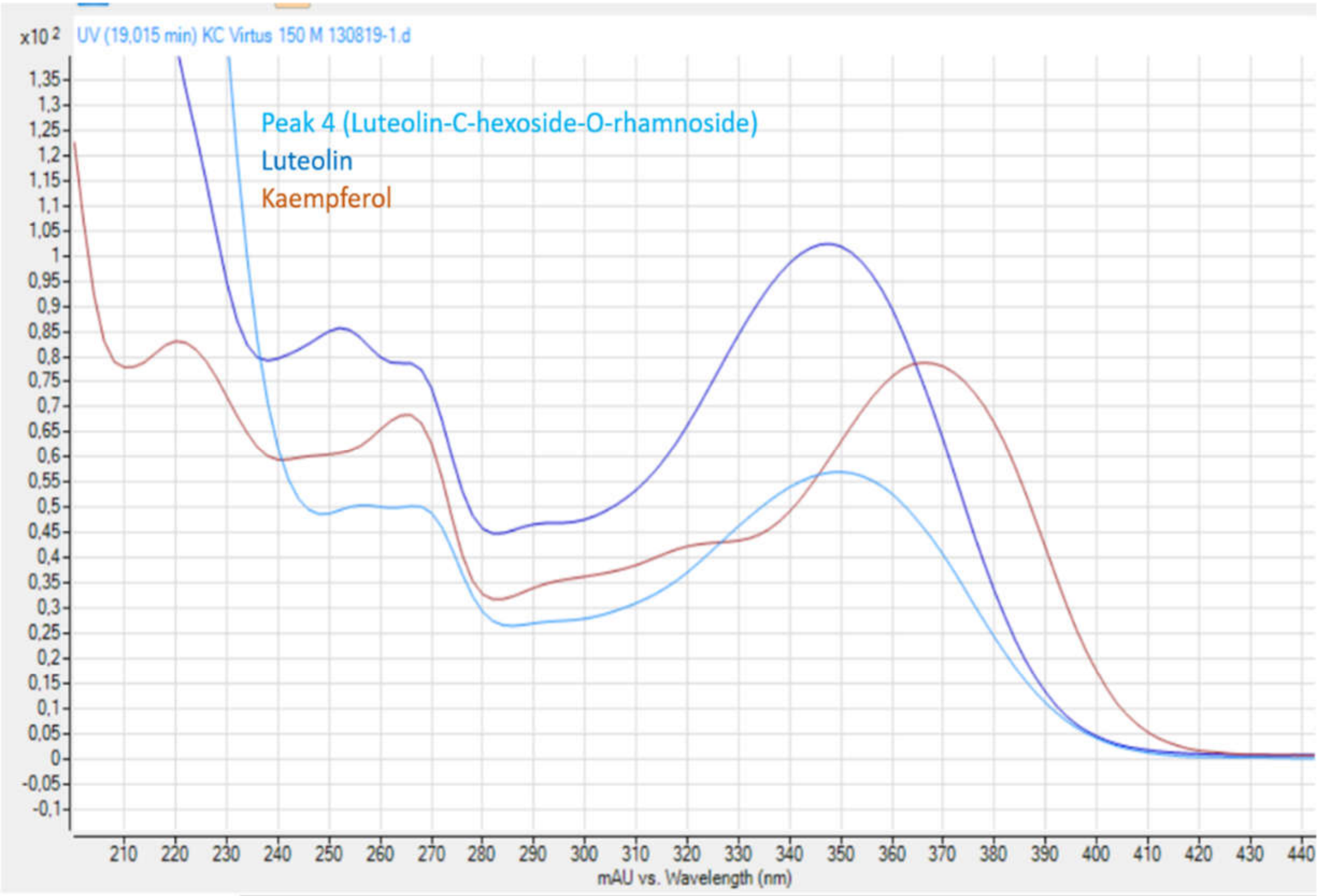

Figure 11. UV spectra of compound **4** (luteolin-C-hexoside-O-rhamnoside), in comparison with UV spectra of Luteolin (blue) and Kaempferol (brown).

Table 4. Flavonoid composition of Fedora 17 density 150. Data are presented as mean ± SD in mg/g DW.

No.	Attempted identification	**22.07.2019** *Early flowering*	**13.08.2019** *Full flowering*	**28.08.2019** *End of flowering*	**10.09.2019** *End of flowering*
1	Quercetin-O-sophoroside	*nd*	*nd*	*nd*	*nd*
2	Isoorientin-2''-O-glucoside	0.386 ± 0.011	0.125 ± 0.002	0.139 ± 0.006	0.071 ± 0.002
3	Luteolin-C-glucoside	0.124 ± 0.001	0.041 ± 0.001	0.026 ± 0.000	0.014 ± 0.002
4	Luteolin-C-hexoside-O-rhamnoside	0.249 ± 0.001	0.111 ± 0.001	0.110 ± 0.004	0.056 ± 0.003
5	Kaempferol-O-sophoroside	*nd*	*nd*	*nd*	*nd*
6	Vitexin-2''-O-glucoside	0.329 ± 0.004	0.062 ± 0.005	0.048 ± 0.006	0.018 ± 0.003
7	Vitexin-2-O-rhamnoside *	0.279 ± 0.014	0.082 ± 0.006	0.056 ± 0.007	0.014 ± 0.007
8	Luteolin-7-*O*-glucuronide *	1.836 ± 0.080	0.830 ± 0.005	0.713 ± 0.022	0.396 ± 0.007
9	Apigenin-7-*O*-glucuronide *	0.242 ± 0.007	0.123 ± 0.007	0.074 ± 0.009	0.049 ± 0.006
10	Chrysoeriol-O-glucuronide isomer 1	0.068 ± 0.001	0.039 ± 0.002	0.030 ± 0.002	0.026 ± 0.004
11	Chrysoeriol-O-glucuronide isomer 2	*nd*	*nd*	*nd*	*nd*
12	Apigenin-4'-methoxy-7-glucuronide	*nd*	0.010 ± 0.000	0.001 ± 0.001	*nd*
13	Luteolin *	*nd*	*Nd*	*nd*	*nd*
14	Apigenin *	*nd*	*nd*	*nd*	*nd*
15	Chrysoeriol *	*nd*	*nd*	*nd*	*nd*
	Total amount of identified flavonoids	3.513 ± 0.119	1.425 ± 0.029	1.198 ± 0.056	0.644 ± 0.020

nd not detected.

Table 5. Flavonoid composition of Felina 32 density 300 and 150 seeds/m^2. Data are presented as mean ± SD in mg/g DW.

No	Attempted identification	Density 150				Density 300	
		**22.07.2019** *Early flowering*	**13.08.2019** *Full flowering*	**27.08.2019** *End of flowering*	**10.09.2019** *End of flowering*	**22.07.2019** *Early flowering*	**10.09.2019** *End of flowering*
1	Quercetin-O-sophoroside	nd	nd	nd	nd	nd	nd
2	Isoorientin-2''-O-glucoside	0.166 ± 0.009	0.095 ± 0.004	0.112 ± 0.002	0.018 ± 0.004	0.514 ± 0.005	0.105 ± 0.003
3	Luteolin-C-glucoside	0.073 ± 0.004	0.021 ± 0.001	0.023 ± 0.002	0.001 ± 0.002	0.208 ± 0.002	0.023 ± 0.001
4	Luteolin-C-hexoside-O-rhamnoside	0.186 ± 0.008	0.111 ± 0.003	0.123 ± 0.008	0.030 ± 0.006	0.328 ± 0.003	0.098 ± 0.003
5	Kaempferol-O-sophoroside	nd	nd	nd	nd	nd	nd
6	Vitexin-2''-O-glucoside	0.249 ± 0.015	0.134 ± 0.006	0.080 ± 0.002	0 ± 0.006	0.745 ± 0.009	0.060 ± 0.004
7	Vitexin-2-O-rhamnoside *	0.408 ± 0.011	0.217 ± 0.006	0.120 ± 0.008	0.009 ± 0.009	0.629 ± 0.009	0.079 ± 0.008
8	Luteolin-7-*O*-glucuronide *	1.983 ± 0.177	1.177 ± 0.050	1.312 ± 0.003	0.535 ± 0.086	3.381 ± 0.032	0.977 ± 0.034
9	Apigenin-7-*O*-glucuronide *	0.641 ± 0.029	0.391 ± 0.011	0.284 ± 0.017	0.181 ± 0.029	0.734 ± 0.010	0.181 ± 0.003
10	Chrysoeriol-O-glucuronide isomer 1	0.073 ± 0.001	0.054 ± 0.001	0.049 ± 0.003	0.038 ± 0.009	0.101 ± 0.001	0.036 ± 0.002
11	Chrysoeriol-O-glucuronide isomer 2	nd	nd	nd	nd	nd	nd
12	Apigenin-4'-methoxy-7-glucuronide	0.641 ± 0.001	0.391 ± 0.000	0.284 ± 0.001	0.181 ± 0.029	0.734 ± 0.001	0.181 ± 0.001
13	Luteolin *	nd	nd	nd	nd	nd	nd
14	Apigenin *	nd	nd	nd	nd	nd	nd
15	Chrysoeriol *	nd	nd	nd	nd	nd	nd
	Total amount of identified flavonoids	4.420 ± 0.251	2.591 ± 0.084	2.388 ± 0.006	0.950 ± 0.145	7.373 ± 0.075	1.741 ± 0.059

nd: not detected.

Table 6. Flavonoid composition of KC Virtus density 30 seeds/m^{2,} male and female inflorescences. Data are presented as mean ± SD in mg/g DW.

No.	Attempted identification	Density 30 Male flowers	Density 30 Female flowers	
		27.08.2019	27.08.2019 *Early flowering*	10.09.19 *Full flowering*
1	Quercetin-*O*-sophoroside	0.175 ± 0.009	ND	ND
2	Isoorientin-2''-O-glucoside	0.157 ± 0.001	0.453 ± 0.038	0.153 ± 0.003
3	Luteolin-C-glucoside	0.044 ± 0.000	0.175 ± 0.012	0.043 ± 0.002
4	Luteolin-C-hexoside-*O*-rhamnoside	0.094 ± 0.000	0.259 ± 0.021	0.144 ± 0.000
5	Kaempferol-*O*-sophoroside	0.070 ± 0.003	ND	ND
6	Vitexin-2''-*O*-glucoside	0.265 ± 0.011	0.719 ± 0.063	0.165 ± 0.006
7	Vitexin-2-*O*-rhamnoside *	0.189 ± 0.001	0.468 ± 0.032	0.166 ± 0.006
8	Luteolin-7-*O*-glucuronide *	1.261 ± 0.014	2.510 ± 0.201	1.675 ± 0.031
9	Apigenin-7-*O*-glucuronide *	0.315 ± 0.001	0.483 ± 0.034	0.392 ± 0.002
10	Chrysoeriol-*O*-glucuronide isomer 1	0.089 ± 0.001	0.098 ± 0.007	0.101 ± 0.003
11	Chrysoeriol-*O*-glucuronide isomer 2	0.092 ± 0.003	0.071 ± 0.005	ND
12	Apigenin-4'-methoxy-7-glucuronide	ND	ND	ND
13	Luteolin *	ND	ND	ND
14	Apigenin *	ND	ND	ND
15	Chrysoeriol *	ND	ND	ND
	Total amount of identified flavonoids	2.751	5.235	2.838

nd: not detected.

Table 7. Flavonoid composition of KC Virtus density 150 seeds/m^{2,} male and female inflorescences. Data are presented as mean ± SD in mg/g DW.

No.	Attempted identification	Density 150 Male flowers		Density 150 Female flowers			
		13.08.2019	27.08.2019	22.07.2019 *Early flowering*	13.08.2019 *Early flowering*	27.08.2019 *Early flowering*	10.09.2019 *Full flowering*
1	Quercetin-*O*-sophorosid	0.043 ± 0.005	0.213 ± 0.002	ND	ND	ND	ND
2	Isoorientin-2''-*O*-glucoside	0.051 ± 0.003	0.236 ± 0.008	0.302 ± 0.023	0.219 ± 0.015	0.232 ± 0.002	0.034 ± 0.048
3	Luteolin-*C*-glucoside	0.020 ± 0.000	0.055 ± 0.002	0.155 ± 0.010	0.065 ± 0.004	0.062 ± 0.001	0.004 ± 0.006
4	Luteolin-C-hexoside-*O*-rhamnoside	0.052 ± 0.003	0.179 ± 0.006	0.160 ± 0.012	0.174 ± 0.011	0.144 ± 0.003	0.031 ± 0.044
5	Kaempferol-*O*-sophorosid	0.029 ± 0.000	0.059 ± 0.001	ND	ND	ND	ND
6	Vitexin-2''-*O*-glucoside	0.061 ± 0.007	0.440 ± 0.021	0.841 ± 0.010	0.487 ± 0.033	0.400 ± 0.009	0.0430.061
7	Vitexin-2-*O*-rhamnoside *	0.153 ± 0.010	0.405 ± 0.018	0.558 ± 0.156	0.433 ± 0.023	0.246 ± 0.005	< 0.075
8	Luteolin-7-*O*-glucuronid *	0.548 ± 0.018	1.544 ± 0.052	2.424 ± 0.161	2.053 ± 0.161	1.165 ± 0.007	0.259 ± 0.325
9	Apigenin-7-*O*-glucuronid *	0.144 ± 0.002	0.393 ± 0.017	0.882 ± 0.031	0.696 ± 0.039	0.281 ± 0.000	0.102 ± 0.075
10	Chrysoeriol-*O*-glucuronid isomer 1	0.032 ± 0.001	0.070 ± 0.003	0.105 ± 0.002	0.082 ± 0.004	0.054 ± 0.001	0.022 ± 0.003
11	Chrysoeriol-*O*-glucuronid isomer 2	0.046 ± 0.003	0.093 ± 0.008	0.118 ± 0.003	0.119 ± 0.008	0.047 ± 0.000	0.026 ± 0.013
12	Apigenin-4'-methoxy-7-glucuronid	ND	ND	0.033 ± 0.002	0.057 ± 0.003	ND	ND
13	Luteolin *	0.118 ± 0.005	ND	ND	ND	ND	ND
14	Apigenin *	0.07 ± 70.002	ND	ND	ND	ND	ND
15	Chrysoeriol *	0.018 ± 0.000	ND	ND	ND	ND	ND
	Total amount of identified flavonoids	1.399	3.694	5.584	4.390	2.635	0.514

nd: not detected.

Table 8. Flavonoid composition of KC Virtus density 300 seeds/m[2,] male and female inflorescences. Data are presented as mean ± SD in mg/g DW.

No.	Attempted identification	Density 300 Male flowers		Density 300 Female flowers			
		13.08.2019	27.08.2019	22.07.2019 *Early flowering*	13.08.2019 *Early flowering*	27.08.2019 *Early flowering*	10.09.2019 *Full flowering*
1	Quercetin-*O*-sophorosid	0.271 ± 0.026	0.055 ± 0.003	ND	ND	ND	ND
2	Isoorientin-2''-*O*-glucoside	0.356 ± 0.019	0.088 ± 0.002	0.228 ± 0.010	0.382 ± 0.013	0.270 ± 0.005	0.028*
3	Luteolin-*C*-glucoside	0.113 ± 0.004	0.030 ± 0.001	0.114 ± 0.006	0.141 ± 0.005	0.079 ± 0.002	ND
4	Luteolin-*C*-hexoside-*O*-rhamnoside	0.193 ± 0.010	0.058 ± 0.001	0.159 ± 0.007	0.223 ± 0.009	0.142 ± 0.001	0.029*
5	Kaempferol-*O*-sophorosid	0.128 ± 0.008	0.028 ± 0.000	ND	ND	ND	ND
6	Vitexin-2''-*O*-glucoside	0.640 ± 0.025	0.210 ± 0.004	0.519 ± 0.023	1.015 ± 0.008	0.431 ± 0.006	0.002*
7	Vitexin-2-*O*-rhamnoside *	0.441 ± 0.013	0.221 ± 0.005	0.503 ± 0.014	0.697 ± 0.012	0.265 ± 0.006	0.021*
8	Luteolin-7-*O*-glucuronid *	2.175 ± 0.119	0.524 ± 0.003	2.674 ± 0.081	2.390 ± 0.061	1.485 ± 0.035	0.396*
9	Apigenin-7-*O*-glucuronid *	0.519 ± 0.011	0.265 ± 0.001	0.947 ± 0.017	0.858 ± 0.000	0.365 ± 0.012	0.117 *
10	Chrysoeriol-*O*-glucuronid isomer 1	0.091 ± 0.001	0.054 ± 0.000	0.121 ± 0.004	0.071 ± 0.002	0.097 ± 0.001	ND
11	Chrysoeriol-*O*-glucuronid isomer 2	0.105 ± 0.001	0.061 ± 0.000	0.115 ± 0.005	0.157 ± 0.006		ND
12	Apigenin-4'-methoxy-7-glucuronid	ND	ND	0.037 ± 0.002	0.014 ± 0.000	0.016 ± 0.001	0.019*
13	Luteolin *	0.003 ± 0.004	0.053 ± 0.001	ND	ND	ND	ND
14	Apigenin *	0.043 ± 0.002	0.033 ± 0.000	ND	ND	ND	ND
15	Chrysoeriol *	ND	ND	ND	ND	ND	ND
	Total amount of identified flavonoids	5.083	1.685	5.424	5.953	3.154	0.673*

* No standard deviation could be calculated; nd: not detected.

Table 9. Flavonoid composition of Fibror 79 density 300, 150 and 30 seeds/m[2] inflorescences. Data are presented as mean ± SD in mg/g DW.

**No.**	**Attempted identification**	**Density 300**			**Density 150**				**Density 30**		
		**22.07.2019** *Early flowering*	**27.08.2019** *Early flowering*	**10.09.2019** *Full flowering*	**22.07.2019** *Early flowering*	**13.08.2019** *Early flowering*	**27.08.2019** *Early flowering*	**10.09.2019** *Full flowering*	**13.08.2019** *Early flowering*	**27.08.2019** *Early flowering*	**10.09.2019** *Full flowering*
1	Quercetin-*O*-sophoroside	nd	nd	nd	nd	nd	nd	nd	nd	nd	nd
2	Isoorientin-2''-*O*-glucoside	0.234 ± 0.011	0.082 ± 0.012	0.006 ± 0.001	0.275 ± 0.014	0.183 ± 0.017	0.052 ± 0.000	0.094 ± 0.002	0.216 ± 0.002	0.183 ± 0.009	0.124 ± 0.010
3	Luteolin-*C*-glucoside	0.092 ± 0.003	0.028 ± 0.003	0.000	0.107 ± 0.002	0.035 ± 0.010	0.011 ± 0.001	0.011 ± 0.003	0.051 ± 0.003	0.035 ± 0.002	0.022 ± 0.002
4	Luteolin-*C*-hexoside-*O*-rhamnoside	0.176 ± 0.004	0.059 ± 0.008	0.005 ± 0.000	0.216 ± 0.005	0.126 ± 0.019	0.043 ± 0.001	0.073 ± 0.004	0.206 ± 0.001	0.126 ± 0.007	0.072 ± 0.004
5	Kaempferol-*O*-sophoroside	nd	nd	nd	nd	nd	nd	nd	nd	nd	nd
6	Vitexin-2''-*O*-glucoside	0.621 ± 0.028	0.147 ± 0.022	0.000	0.697 ± 0.046	0.267 ± 0.042	0.115 ± 0.005	0.119 ± 0.007	0.360 ± 0.001	0.267 ± 0.016	0.072 ± 0.009
7	Vitexin-2-*O*-rhamnoside *	0.573 ± 0.017	0.152 ± 0.018	0.003 ± 0.007	0.704 ± 0.034	0.176 ± 0.018	0.126 ± 0.004	0.081 ± 0.003	0.377 ± 0.001	0.176 ± 0.011	0.019 ± 0.005
8	Luteolin-7-*O*-glucuronide *	2.669 ± 0.233	0.917 ± 0.119	0.111 ± 0.023	2.691 ± 0.176	2.035 ± 0.136	0.673 ± 0.000	0.761 ± 0.025	2.077 ± 0.011	2.035 ± 0.196	1.024 ± 0.095
9	Apigenin-7-*O*-glucuronide *	1.132 ± 0.046	0.419 ± 0.032	0.152 ± 0.040	1.065 ± 0.040	0.582 ± 0.009	0.367 ± 0.003	0.269 ± 0.001	0.578 ± 0.008	0.582 ± 0.050	0.256 ± 0.003
10	Chrysoeriol-*O*-glucuronide isomer 1	0.093 ± 0.004	0.054 ± 0.005	0.013 ± 0.001	0.143 ± 0.007	0.077 ± 0.002	0.076 ± 0.000	0.051 ± 0.004	0.111 ± 0.000	0.077 ± 0.003	0.042 ± 0.004
11	Chrysoeriol-*O*-glucuronide isomer 2	0.061 ± 0.012	0.026 ± 0.002	0.021 ± 0.002	nd	nd	nd	nd	nd	nd	nd
12	Apigenin-4'-methoxy-7-glucuronide	0.058 ± 0.001	0.000	0.010 ± 0.004	0.019 ± 0.000	nd	0.011 ± 0.003	0.024 ± 0.001	0.031 ± 0.000	nd	nd
13	Luteolin *	nd	nd	nd	nd	nd	nd	nd	nd	nd	nd

14	Apigenin *	nd	nd	nd	nd	nd	nd	nd	nd	nd	nd
15	Chrysoeriol *	nd	nd	nd	nd	nd	nd	nd	nd	nd	nd
	Total amount of identified flavonoids	5.71	1.89	0.30	5.92	3.49	1.48	1.49	4.01	3.49	1.63

nd: not detected.

Table 10. Flavonoid composition of Santhica 27 density 300 seeds/m^2 inflorescences. Data are presented as mean ± SD in mg/g DW.

No.	Attempted identification	22.07.2019 *Early flowering*	13.08.2019 *Full flowering*	27.08.2019 *Full flowering*	10.09.2019 *End of flowering*
1	Quercetin-*O*-sophoroside	nd	nd	nd	nd
2	Isoorientin-2''-*O*-glucoside	0.175 ± 0.001	0.134 ± 0.000	0.000	0.000
3	Luteolin-*C*-glucoside	0.075 ± 0.000	0.031 ± 0.000	0.000	0.000
4	Luteolin-*C*-hexoside-*O*-rhamnoside	0.228 ± 0.001	0.155 ± 0.000	0.004 ± 0.001	0.004 ± 0.000
5	Kaempferol-*O*-sophoroside	nd	nd	nd	nd
6	Vitexin-2''-O-glucoside	0.232 ± 0.001	0.154 ± 0.001	0.000	0.000
7	Vitexin-2-*O*-rhamnoside *	0.528 ± 0.002	0.250 ± 0.001	0.000	0.000
8	Luteolin-7-*O*-glucuronide *	1.896 ± 0.021	1.216 ± 0.080	0.061 ± 0.010	0.060 ± 0.005
9	Apigenin-7-*O*-glucuronide *	0.688 ± 0.001	0.256 ± 0.004	0.056 ± 0.012	0.094 ± 0.012
10	Chrysoeriol-*O*-glucuronide isomer 1	0.077 ± 0.012	0.039 ± 0.002	0.016 ± 0.000	0.024 ± 0.000
11	Chrysoeriol-*O*-glucuronide isomer 2	nd	nd	nd	nd
12	Apigenin-4'-methoxy-7-glucuronide	0.065 ± 0.003	0.026 ± 0.000	0.004 ± 0.001	0.004 ± 0.003
13	Luteolin *	nd	nd	nd	nd
14	Apigenin *	nd	nd	nd	nd
15	Chrysoeriol *	nd	nd	nd	nd
	Total amount of identified flavonoids	3.97	2.26	0.14	0.19

nd: not detected.

Table 11. Flavonoid composition of Futura 75 density 300 and 150 seeds/m² inflorescences. Data are presented as mean ± SD in mg/g DW.

No.	Attempted identification	Density 150				Density 300		
		**22.07.2019** *Early flowering*	**13.08.2019** *Early flowering*	**27.08.2019** *Full flowering*	**10.09.2019** *End of flowering*	**22.07.2019** *Early flowering*	**13.08.2019** *Early flowering*	**10.09.2019** *End of flowering*
1	Quercetin-*O*-sophoroside	nd	0.014 ± 0.002	nd	nd	nd	nd	nd
2	Isoorientin-2''-*O*-glucoside	0.036 ± 0.004	0.105 ± 0.008	0.191 ± 0.001	0.177 ± 0.000	0.175 ± 0.000	0.007 ± 0.001	0.060 ± 0.003
3	Luteolin-*C*-glucoside	0.031 ± 0.003	0.034 ± 0.002	0.008 ± 0.020	0.022 ± 0.002	0.055 ± 0.000	0.001 ± 0.000	0.009 ± 0.002
4	Luteolin-C-hexoside-*O*-rhamnoside	0.087 ± 0.008	0.103 ± 0.005	0.132 ± 0.013	0.124 ± 0.003	0.164 ± 0.001	0.007 ± 0.001	0.040 ± 0.001
5	Kaempferol-*O*-sophoroside	nd	0.019 ± 0.003	nd	nd	nd	nd	nd
6	Vitexin-2''-*O*-glucoside	0.000	0.105 ± 0.010	0.273 ± 0.008	0.166 ± 0.005	0.304 ± 0.012	0.079 ± 0.006	0.061 ± 0.007
7	Vitexin-2-*O*-rhamnoside *	0.178 ± 0.017	0.152 ± 0.014	0.171 ± 0.005	0.099 ± 0.003	0.382 ± 0.002	0.122 ± 0.004	0.055 ±0.012
8	Luteolin-7-*O*-glucuronide *	1.915 ± 0.204	1.116 ± 0.079	1.404 ± 0.195	1.220 ± 0.135	2.049 ± 0.056	0.160 ± 0.008	0.578 ± 0.011
9	Apigenin-7-*O*-glucuronide *	0.766 ± 0.047	0.261 ± 0.033	0.334 ± 0.022	0.203 ± 0.008	0.780 ± 0.002	0.313 ± 0.011	0.240 ± 0.014
10	Chrysoeriol-*O*-glucuronide isomer 1	0.064 ± 0.004	0.036 ± 0.003	0.038 ± 0.001	0.032 ± 0.000	0.060 ± 0.000	0.027 ± 0.000	0.028 ± 0.001
11	Chrysoeriol-*O*-glucuronide isomer 2	0.191 ± 0.009	0.065 ± 0.004	0.067 ± 0.000	0.045 ± 0.000	0.105 ± 0.000	0.042 ± 0.000	0.056 ± 0.003
12	Apigenin-4'-methoxy-7-glucuronide	nd	nd	nd	nd	nd	nd	nd
13	Luteolin *	nd	0.039 ± 0.002	nd	nd	nd	nd	nd
14	Apigenin *	nd	0.018 ± 0.001	nd	nd	nd	nd	nd
15	Chrysoeriol *	nd	nd	nd	nd	nd	nd	nd
	Total amount of identified flavonoids	3.27	2.07	2.62	2.09	4.07	0.76	1.13

nd: not detected.

Table 12. Flavonoid composition of Santhica 70 density 300 seeds/m^2 inflorescences. Data are presented as mean ± SD in mg/g DW.

No.	Attempted identification	22.07.2019 *Early flowering*	13.08.2019 *Full flowering*	10.09.2019 *End of flowering*
1	Quercetin-*O*-sophoroside	nd	nd	nd
2	Isoorientin-2''-*O*-glucoside	0.252 ± 0.006	0.013 ± 0.000	0.000
3	Luteolin-*C*-glucoside	0.075 ± 0.003	0.004 ± 0.000	0.000
4	Luteolin-C-hexoside-*O*-rhamnoside	0.282 ± 0.007	0.027 ± 0.001	0.000
5	Kaempferol-*O*-sophoroside	nd	nd	nd
6	Vitexin-2''-*O*-glucoside	0.406 ± 0.002	0.000	0.000
7	Vitexin-2-*O*-rhamnoside *	0.596 ± 0.014	0.071 ± 0.000	0.000
8	Luteolin-7-*O*-glucuronide *	2.474 ± 0.050	0.306 ± 0.023	0.005 ± 0.009
9	Apigenin-7-*O*-glucuronide *	0.648 ± 0.016	0.160 ± 0.000	0.033 ± 0.018
10	Chrysoeriol-*O*-glucuronide isomer 1	0.073 ± 0.005	0.023 ± 0.001	0.013 ± 0.001
11	Chrysoeriol-*O*-glucuronide isomer 2	nd	nd	nd
12	Apigenin-4'-methoxy-7-glucuronide	0.036 ± 0.001	0.010 ± 0.001	0.005 ± 0.000
13	Luteolin *	nd	nd	nd
14	Apigenin *	nd	nd	nd
15	Chrysoeriol *	nd	nd	nd
	Total amount of identified flavonoids	4.84	0.61	0.06

Table 13. Flavonoid composition of Finola density 300 and 150 seeds/m^2, male and female inflorescences. Data are presented as mean ± SD in mg/g DW.

No.	Attempted identification	Density 150					Density 300
		Male flowers	Female flowers				
		02.07.2019	02.07.2019 *Full flowering*	16.07.2019 *End of flowering*	30.07.2019 *End of flowering*	11.08.2019 *End of flowering*	11.08.2019 *End of flowering*
1	Quercetin-*O*-sophorosid	0.059 ± 0.004	nd	nd	nd	nd	nd
2	Isoorientin-2''-*O*-glucoside	0.172 ± 0.003	0.205± 0.008	0.129 ± 0.001	0.089 ± 0.002	0.059 ± 0.002	0.025 ± 0.002
3	Luteolin-*C*-glucoside	0.049 ± 0.001	0.117 ± 0.000	0.052 ± 0.000	0.028 ± 0.001	0.008 ± 0.000	0.012 ± 0.001
4	Luteolin-*C*-hexoside-*O*-rhamnoside	0.149 ± 0.001	0.182 ± 0.003	0.109 ± 0.001	0.072 ± 0.003	0.054 ± 0.001	0.030 ± 0.002
5	Kaempferol-*O*-sophorosid	0.033 ± 0.001	Nd	Nd	Nd	Nd	nd
6	Vitexin-2''-*O*-glucoside	0.125 ± 0.005	0.167 ± 0.006	0.093 ± 0.001	0.011 ± 0.002	Nd	Nd
7	Vitexin-2-*O*-rhamnoside *	0.126 ± 0.004	0.250 ± 0.015	0.101 ± 0.026	0.017 ± 0.004	Nd	Nd
8	Luteolin-7-*O*-glucuronid *	1.019 ± 0.005	1.416 ± 0.007	1.035 ± 0.008	0.630 ± 0.046	0.322 ± 0.063	0.165 ± 0.013
9	Apigenin-7-*O*-glucuronid *	0.131 ± 0.002	0.257 ± 0.002	0.175 ± 0.002	0.078 ± 0.004	0.019 ± 0.002	0.050 ± 0.005
10	Chrysoeriol-*O*-glucuronid isomer 1	0.069 ± 0.002	0.059 ± 0.000	0.049 ± 0.001	0.033 ± 0.005	0.021 ± 0.001	0.031 ± 0.001
11	Chrysoeriol-*O*-glucuronid isomer 2	nd	nd	nd	nd	nd	nd
12	Apigenin-4'-methoxy-7-glucuronid	nd	nd	nd	nd	nd	nd
13	Luteolin *	nd	nd	nd	nd	nd	nd
14	Apigenin *	nd	nd	nd	nd	nd	nd
15	Chrysoeriol *	nd	nd	nd	nd	nd	nd
	Total amount of identified flavonoids	1.931	2.654	1.742	0.958	2.351	0.263

Table 14. Qualitative analysis of the different terpenes found in 8 different hemp varieties.

Cultivar, sowing density, harvest date	Camphene [a]	alpha-Pinene [b]	beta-Pinene [b]	3-Caren [b]	alpha-Phellandrene [b]	beta-Myrcene [b]	Limonene [b]	Eucalyptol [b]	beta-Phellandrene [a]	(Z)-beta-Ocimene [b]	gamma-Terpinene [b]	(E)-beta-Ocimene [b]	4-Cymen [b]	Terpinolene [b]	Linolool [b]	alpha-Santalene [a]	(E)-alpha-Bergamotene [a]	(E)-beta-Caryophyllene [b]	9-epi-(E)-Caryophylllene	alpha-Humulene [b]	(E)-beta-Farnesene [a]	beta-Selinene [a]	alpha-Selinene [a]	gamma-Cadinene [a]	beta-Maaliene [a]	Selina-3,7(11)-diene [a]	beta-(E)-	Humulen-1,2-epoxid [a]	alpha-Bisabolol [b]
Fedora 17 150, 27.08.19 (*end of flowering)*	+++	++++	++++	++++	+++	+++++	+++	+++	+++	+++	+++	++++	+++	+++++	+	++	+++	++++	++	++++	+++	+++	+++	++	++	+++	++++	+++	+++
Santhica 70 300, 10.09.19 (*end of flowering*)	++	++++	+++	++	++	++++	+++	+++	+++	++	++	+++	++	++++	+	++	+++	++++	++	++++	+++	+++	+++	++	++	+++	+++	+++	++
Finola 150, 11.08.19 (*end of flowering*)	+++	++++	++++	+++	++	++++	+++	+++	+++	+++	++	++++	++	++++	+	++	+++	++++	++	+++	+++	++	++	++	++	++	+++	+++	+++
Fibror 79 150, 27.08.19 (*end of early flowering*)	+++	++++	++++	++++	±	+++++	++++	+++	+++	+++	++	++++	+++	++++	++	++	+++	++++	+++	++++	++++	+++	+++	+++	+++	+++	++++	+++	+++
Felina 32 150, 27.08.19 (*end of flowering*)	+++	++++	++++	+++	+++	+++++	+++	+++	++++	+++	+++	++++	+++	+++++	++	+++	++++	++++	+++	++++	++++	+++	+++	++	++	++	++++	+++	++
Santhica 27 300, 10.09.19 (*end of flowering*)	++	++++	+++	++	++	++++	+++	+++	++	++	++	+++	++	+++	+	++	+++	++++	++	++++	+++	+++	++	++	++	++	+++	+++	++
Futura 75 150, 27.08.19 (*full flowering*)	+++	+++++	++++	+++	+++	+++++	++++	+++	++++	+++	+++	++++	+++	+++++	++	+++	++++	++++	+++	++++	++++	+++	+++	++	+++	+++	++++	+++	+++
KC Virtus ♂ 300, 27.08.19	±	++++	+++	±	±	++++	+++	+++	+++	++	±	+++	±	+++	±	++	±	++++	±	+++	±	+++	++	±	++	±	+++	+++	+++

KC Virtus 300, 27.08.19 (*end of early flowering*)	+++	++++	++++	+++	++	+++++	++++	+++	+++	+++	++	+++	+++	++++	+	++	+++	++++	++	++++	+++	+++	+++	++	+++	+++	++++	+++	+++

[a] Compound identified by mass and retention index, not verified with reference standard.

[b] Compound identified by mass and retention index, and verified with reference standard.

++++, +++, ++, +, ± relative amount of each compounds in the sample calculated using the formula n+ = log($A_{Scan\ 35..250\ AMU}$) - 4.

Table 15. Terpene composition of different hemp cultivars sowed at different densities and harvested at different dates. Data are expressed as mean ± SD in mg/kg FW, flowering stages are indicated by the letters: a = early flowering, b = full flowering, c = end of flowering.

**Cultivar, sowing density**	**Harvest date and flowering stage**	*alpha-Pinene*	*beta-Pinene*	*Myrcene*	*Limonene*	*beta-Phellandrene*	*(Z)-Ocimene*	*(E)-Ocimene*	*Eucalyptol*	*gamma-Terpinene*	*Terpinolene*	*Linalool*	*beta-Caryophyllene*	*alpha-Humulene*	*alpha-Bisabolol*	*Sum* [illegible]	*Sum Sesquiterpenes*	*Sum Terpenes*	*Ratio Mono/Sesquiterp*
		**Quantity (mg/kg)**																	
Fedora 17 150	22.07.2019 a	50.8 ± 5.4	19.5 ± 1.5	17.1 ± 1.6	4.0 ± 0.5	5.6 ± 0.5	2.2 ± 0.5	16.9 ± 1.1	2.1 ± 0.5	< 0.5	6.7 ± 1.1	< 0.5	220.3 ± 10.0	74.1 ± 3.6	6.6 ± 1.7	125.1	301.1	426	0.4
	13.08.2019 b	110.2 ± 1.0	42.4 ± 4.9	111.7 ± 17.5	6.7 ± 1.4	8.7 ± 1.1	5.4 ± 0.9	78.7 ± 12.6	0.6 ± 0.3	1.8 ± 0.5	39.9 ± 4.5	0.9 ± 0.3	169.0 ± 3.6	61.2 ± 1.5	3.0 ± 0.3	407.0	233.2	640	1.7
	27.08.2019 c	140.7 ± 0.5	53.0 ± 1.1	265.9 ± 31.7	13.6 ± 2.2	21.5 ± 1.6	9.1 ± 0.7	135.6 ± 9.3	1.0 ± 0.3	4.1 ± 0.5	168.8 ± 1.7	0.8 ± 0.2	182.2 ± 13.3	59.2 ± 2.7	5.8 ± 0.3	814.2	247.2	1061	3.3
	10.09.2019 c	63.5 ± 1.2	24.4 ± 2.2	162.9 ± 5.6	6.2 ± 0.9	9.9 ± 0.7	3.9 ± 0.7	55.7 ± 3.9	0.9 ± 0.2	1.8 ± 0.3	53.5 ± 1.8	0.8 ± 0.2	62.0 ± 5.2	20.1 ± 1.6	5.2 ± 0.9	383.4	87.3	471	4.4
KC Virtus 150 ☿	22.07.2019 a	39.1 ± 0.3	15.1 ± 2.4	27.8 ± 4.2	9.3 ± 0.5	10.0 ± 0.9	0.7 ± 0.3	4.5 ± 0.3	6.5 ± 0.3	< 0.5	< 0.5	< 0.5	253.6 ± 64.0	79.0 ± 21.4	13.1 ± 0.3	114.6	345.7	460	0.3
	13.08.2019 a	91.2 ± 0.5	49.2 ± 10.2	77.5 ± 19.3	23.1 ± 7.1	14.2 ± 2.7	1.3 ± 0.9	11.8 ± 3.5	3.4 ± 0.3	0.9 ± 0.2	7.2 ± 2.2	< 0.5	324.7 ± 62.5	106.3 ± 20.1	3.7 ± 0.2	280.5	434.6	715	0.6
	27.08.2019 a	112.9 ± 0.5	52.1 ± 1.0	195.8 ± 30.9	22.6 ± 9.0	12.0 ± 1.7	2.6 ± 2.0	18.5 ± 2.7	2.3 ± 0.3	1.0 ± 0.5	42.1 ± 5.9	1.0 ± 0.6	166.2 ± 17.5	51.4 ± 4.8	4.7 ± 0.4	462.9	222.3	685	2.1
	10.09.2019 b	127.4 ± 0.6	54.2 ± 3.8	214.7 ± 22.5	32.4 ± 1.4	9.6 ± 0.5	2.4 ± 0.3	34.5 ± 2.9	1.4 ± 0.3	0.7 ± 0.2	42.1 ± 4.4	2.5 ± 0.3	158.8 ± 23.8	49.1 ± 7.2	5.7 ± 0.5	521.9	213.6	735	2.4
Finola 150	02.07.2019 b	41.5 ± 10.7	19.9 ± 2.9	36.8 ± 0.6	10.2 ± 0.3	1.1 ± 0.3	2.2 ± 0.8	21.9 ± 0.6	< 0.5	< 0.5	9.0 ± 0.6	0.6 ± 0.7	87.4 ± 6.7	27.0 ± 2.2	5.7 ± 1.4	144.0	120.1	264	1.2
	16.07.2019 c	80.6 ± 4.0	35.2 ± 1.3	146.8 ± 13.9	16.8 ± 1.7	6.8 ± 0.5	3.6 ± 0.7	55.9 ± 5.9	< 0.5	< 0.5	28.9 ± 5.5	0.6 ± 0.5	70.9 ± 2.9	21.6 ± 2.2	3.2 ± 0.3	376.3	95.7	472	3.9
	30.07.2019 c	54.7 ± 3.3	22.6 ± 2.0	117.7 ± 11.2	10.9 ± 0.9	8.1 ± 0.7	3.9 ± 0.7	54.1 ± 6.3	0.8 ± 0.2	1.3 ± 0.4	46.9 ± 3.1	< 0.5	97.0 ± 4.7	29.8 ± 1.9	4.1 ± 0.3	321.6	131.0	453	2.5
	11.08.2019 c	45.3 ± 1.1	18.9 ± 0.7	105.3 ± 2.7	5.0 ± 0.2	7.0 ± 0.3	4.8 ± 0.4	81.6 ± 2.1	0.7 ± 0.2	1.4 ± 0.3	50.5 ± 2.5	0.6 ± 0.5	58.8 ± 3.7	18.3 ± 1.3	5.6 ± 0.7	321.0	82.7	404	3.9
Santhica 70 150	22.07.2019 a	47.9 ± 0.3	14.0 ± 2.7	11.1 ± 1.1	3.3 ± 0.3	4.3 ± 0.2	1.6 ± 0.3	9.3 ± 1.0	3.6 ± 0.2	< 0.5	< 0.5	< 0.5	183.7 ± 45.2	71.9 ± 18.3	5.7 ± 0.2	96.6	261.3	358	0.4
	13.08.2019 b	30.6 ± 0.4	10.2 ± 0.3	32.8 ± 2.9	3.1 ± 0.7	5.5 ± 0.7	1.1 ± 0.5	19.6 ± 1.3	1.7 ± 0.3	1.1 ± 0.5	14.1 ± 0.2	< 0.5	133.0 ± 1.9	53.5 ± 1.5	4.0 ± 0.5	120.3	190.6	311	0.6
	10.09.2019 c	19.5 ± 0.3	7.0 ± 1.0	63.4 ± 10.2	2.4 ± 0.5	4.7 ± 0.5	2.0 ± 0.6	22.5 ± 0.7	2.1 ± 0.4	1.0 ± 0.5	20.9 ± 3.1	< 0.5	73.3 ± 8.1	28.3 ± 2.4	2.8 ± 0.4	146.1	104.4	251	1.4
KC Virtus 150 ⚦	13.08.2019	49.6 ± 0.3	24.2 ± 0.2	94.4 ± 5.5	10.0 ± 1.2	12.1 ± 0.6	1.0 ± 0.3	4.2 ± 0.5	5.7 ± 0.2	< 0.5	1.2 ± 0.5	< 0.5	125.3 ± 11.6	38.7 ± 2.8	5.2 ± 0.2	203.3	169.2	373	1.2

KC Virtus 300 ♂	27.08.2019	30.3 ± 2.4	12.0 ± 2.0	91.4 ± 7.5	4.6 ± 0.7	6.6 ± 1.6	1.1 ± 0.2	5.4 ± 1.0	1.8 ± 1.5	< 0.5	3.6 ± 0.7	< 0.5	83.9 ± 15.4	26.7 ± 1.5	2.3 ± 0.2	157.8	112.9	271	1.4
KC Virtus 300 ♀	22.07.2019 a	39.5 ± 0.4	16.0 ± 1.6	31.7 ± 1.4	11.4 ± 0.8	15.9 ± 0.9	0.9 ± 0.3	5.4 ± 0.8	9.1 ± 0.3	< 0.5 ±	< 0.5	< 0.5	355.1 ± 34.1	109.4 ± 13.9	7.2 ± 0.4	131.4	471.7	603	0.3
	27.08.2019 a	113.0 ± 0.9	55.6 ± 3.9	176.1 ± 22.0	29.2 ± 3.8	17.1 ± 1.4	1.8 ± 0.7	18.2 ± 2.7	4.5 ± 0.3	1.0 ± 0.3	41.4 ± 3.5	1.7 ± 0.7	201.5 ± 6.3	64.1 ± 0.8	11.8 ± 0.3	459.7	277.3	737	1.7
Fibror 79 30	27.08.2019 a	145.3 ± 10.3	56.6 ± 1.2	173.0 ± 0.5	32.1 ± 0.8	8.0 ± 0.3	1.8 ± 0.7	12.7 ± 0.3	1.2 ± 0.2	1.5 ± 0.3	19.2 ± 1.1	1.4 ± 0.7	129.0 ± 3.6	43.6 ± 1.0	3.4 ± 0.4	452.8	176.0	629	2.6
Fibror 79 150	27.08.2019 a	85.1 ± 8.4	43.9 ± 12.4	172.2 ± 18.6	20.4 ± 10.4	10.1 ± 2.5	2.5 ± 0.2	20.6 ± 7.0	1.0 ± 0.2	1.5 ± 0.3	28.7 ± 7.9	1.2 ± 0.8	137.8 ± 15.6	45.0 ± 4.4	6.5 ± 0.8	387.0	189.3	576	2.0
Fibror 79 300	27.08.2019 a	81.2 ± 12.3	32.0 ± 0.9	124.9 ± 3.7	9.0 ± 0.3	6.8 ± 0.3	2.1 ± 0.2	29.2 ± 4.2	0.8 ± 0.1	0.6 ± 0.1	31.8 ± 3.0	0.8 ± 0.6	159.8 ± 29.2	48.4 ± 8.3	6.2 ± 0.4	319.2	214.4	534	1.5
Felina 32 150	27.08.2019 c	101.4 ± 2.0	46.8 ± 1.9	305.6 ± 17.7	6.2 ± 0.6	22.1 ± 1.2	3.5 ± 0.6	60.5 ± 3.4	2.0 ± 0.4	2.5 ± 0.5	133.3 ± 17.5	< 0.5	214.0 ± 11.7	80.9 ± 2.9	3.1 ± 0.3	684.4	298.0	982	2.3
Santhica 27 300	27.08.2019 b	21.5 ± 1.4	6.1 ± 0.4	34.5 ± 3.5	2.2 ± 0.4	4.6 ± 0.4	1.3 ± 0.6	15.4 ± 1.7	1.3 ± 0.3	0.8 ± 0.2	12.7 ± 2.2	< 0.5	90.8 ± 8.6	35.9 ± 2.8	2.9 ± 0.3	100.7	129.6	230	0.8
Futura 75 150	27.08.2019 b	108.5 ± 12.6	47.5 ± 5.0	235.5 ± 44.1	10.6 ± 2.1	21.8 ± 3.3	5.0 ± 0.7	57.9 ± 2.7	2.9 ± 0.2	2.2 ± 0.3	131.5 ± 12.9	1.5 ± 0.8	158.1 ± 21.5	51.0 ± 10.2	5.6 ± 0.3	624.8	214.7	839	2.9
